# Reactive astrocytes as neural stem or progenitor cells: In vivo lineage, In vitro potential, and Genome‐wide expression analysis

**DOI:** 10.1002/glia.22850

**Published:** 2015-05-12

**Authors:** Magdalena Götz, Swetlana Sirko, Johannes Beckers, Martin Irmler

**Affiliations:** ^1^ Physiological GenomicsBiomedical CenterLudwig‐Maximilians‐University MunichMunichGermany; ^2^ Institute of Stem Cell ResearchHelmholtz Center MunichMunichGermany; ^3^ SYNERGY, Excellence Cluster of Systemic NeurologyLMUMunichGermany; ^4^ Institute of Experimental GeneticsHelmholtz Center MunichMunichGermany; ^5^ Department of Experimental GeneticsTechnical University MunichFreising‐WeihenstephanGermany; ^6^ German Center for Diabetes Research (DZD)NeuherbergGermany

**Keywords:** self‐renewal, lineage, potential, radial glial cells, brain injury, transcriptome

## Abstract

Here, we review the stem cell hallmarks of endogenous neural stem cells (NSCs) during development and in some niches of the adult mammalian brain to then compare these with reactive astrocytes acquiring stem cell hallmarks after traumatic and ischemic brain injury. Notably, even endogenous NSCs including the earliest NSCs, the neuroepithelial cells, generate in most cases only a single type of progeny and self‐renew only for a rather short time *in vivo*. *In vitro*, however, especially cells cultured under neurosphere conditions reveal a larger potential and long‐term self‐renewal under the influence of growth factors. This is rather well comparable to reactive astrocytes in the traumatic or ischemic brain some of which acquire neurosphere‐forming capacity including multipotency and long‐term self‐renewal *in vitro*, while they remain within their astrocyte lineage *in vivo*. Both reactive astrocytes and endogenous NSCs exhibit stem cell hallmarks largely *in vitro*, but their lineage differs *in vivo*. Both populations generate largely a single cell type *in vivo*, but endogenous NSCs generate neurons and reactive astrocytes remain in the astrocyte lineage. However, at some early postnatal stages or in some brain regions reactive astrocytes can be released from this fate restriction, demonstrating that they can also enact neurogenesis. Thus, reactive astrocytes and NSCs share many characteristic hallmarks, but also exhibit key differences. This conclusion is further substantiated by genome‐wide expression analysis comparing NSCs at different stages with astrocytes from the intact and injured brain parenchyma. GLIA 2015;63:1452–1468

## Prelude

As we know, the term “stem cell” is among the most disputed definitions and yet everybody knows exactly what it is (Ledford, 2008)—reminiscent of Augustinus' saying about the “time” (Aurelius Augustinus, Confesiones XI, 14). Therefore, let us start with reviewing the definition of stem cells, then review how these criteria apply to the endogenous neural stem cells (NSCs) from development to adulthood to then proceed how these criteria apply to reactive astrocytes. Then, let us move into the genomic area and consider genome‐wide expression pattern of the different NSCs to further understand the differences and similarities between reactive astrocytes and NSCs.

## Introduction

### What Is a Stem Cell?

Stem cells are generally defined as cells that can proliferate in an undifferentiated state without obvious signs of change (self‐renewal), but are also able to form specialized cells (differentiation). The prototype examples of this definition are embryonic stem cells (ESCs) that show a virtual unlimited self‐renewal, but can also give rise to all the cells of the embryo proper and are therefore pluripotent (Nagy et al., 1990). However, albeit the value of ESCs can hardly be overestimated, their limitless expansion *in vitro* is an artifact because their cellular *in vivo* counterparts self‐renew only for a little while as they continuously adopt lineage biases. Thus, ESCs are a prime example of stabilizing a fate *in vitro*, which is rather transient *in vivo* (Wray et al., 2010; Ying et al., 2008). Adult hematopoietic stem cells (HSCs) not only generate all descendants of the blood and immune system but can also self‐renew for so long that they can supply several generations of mice with a full hematopoietic system. However, this has been assessed mostly in regeneration assays using transplanted HSCs, while their *in vivo* lineage has only recently been started to elucidate (Busch et al., 2015). Again, the progeny appears more limited *in vivo*, but certainly comprised still many cell types. There are other stem cells that generate only a single cell type, such as some stem cells in the skin that generate only keratinocytes (Hsu et al., 2014). Similarly, the term self‐renewal becomes disputed when self‐renewal is limited to a few rounds of divisions. So are these still stem cells and what about NSCs? Do they generate all cells of the nervous system and for how long do they self‐renew?

### NSCs During Embryonic Development: Limited Lineage and Self‐Renewal

**Figure 1:**
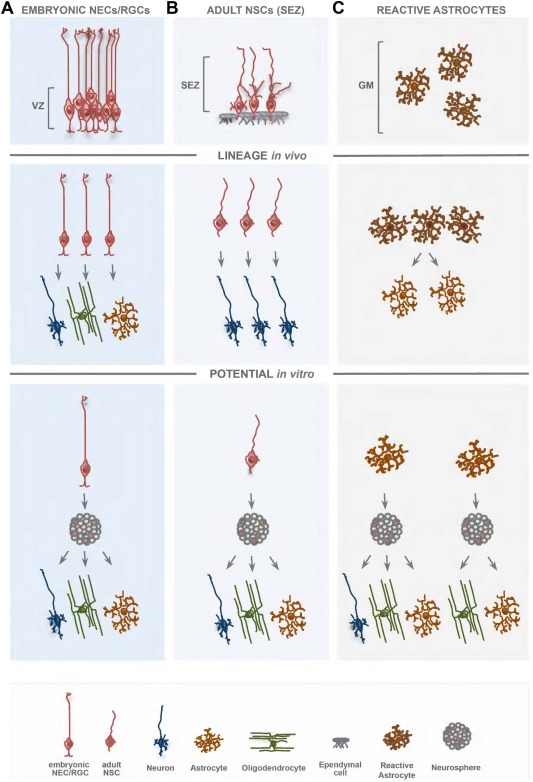
Distinction between *LINEAGE* and *POTENTIAL* of a single embryonic RGC, adult NCS, and proliferating reactive astrocyte from the adult cerebral cortex. Radial glial cells (RGCs) with their main contacts at the apical side and the basement membrane are widespread in the developing vertebrate CNS and persist into adulthood in the highly specialized stem cell niches and are referred to as adult NSCs. Adult NSCs possess radial glia hallmarks, such as apical contact with the ventricle and a shortened basal process. Both RGCs and adult NSCs are able to generate neurons and glia cells, but at the single‐cell level they are largely uni/bilineage *in vivo*. In contrast, the injury‐induced proliferation of parenchymal astrocytes, unlike RGCs/NSCs, resulted in the generation of astrocytes only. Even when proliferating reactive astrocytes are astroglial‐restricted, they show a larger potential when exposed to a different environment *in vitro*, and in similarity to RGCs or NSCs can be instructed to multipotency and long‐term self‐renewal upon exposure to growth factors. VZ, ventricular zone; SEZ, subependymal zone; GM, gray matter of cerebral cortex.

During development of the vertebrate central nervous system (CNS) the first ancestors to appear are the neuroepithelial cells (NECs). They are mostly amplifying the pool of stem and progenitor cells of the CNS initially (Gao et al., 2014) and are certainly at the base of all CNS cell types, including neurons, astrocytes, oligodendrocytes, and ependymal cells (Fig. 1). However, when the progeny of a single cell is monitored *in vivo*, either by dye labeling, viral vectors, or Cre‐mediated fate mapping, in most cases single NECs generate a single cell type, mostly neurons, with only a minority generating two cell types (1 of 6 ≈ 17% in Gao et al., 2014; Guérout et al., 2014; Rowitch and Kriegstein, 2010). An important hallmark of the CNS is its patterning, such that NECs located at different positions express different fate determinants and generate different progeny. For example, expression of the transcription factors Olig2 or Ascl1 in the ventral spinal cord has been shown to regulate the generation of motor neurons first and later the generation of oligodendrocytes (Guérout et al., 2014; Li et al., 2011; Takebayashi and Ikenaka, 2015). However, transplantation experiments, genetic fate mapping, and genetic induction of death of motor neuron progenitors showed that Olig2+ or Ascl1+ motor neuron progenitors are not in the same lineage as the later Olig2+ or Ascl1+ oligodendrocyte progenitor cells (Battiste et al., 2007; Mukouyama et al., 2006; Wu et al., 2006). Thus, even the earliest ancestors of the CNS, the NECs that further share expression of a specific fate determinant transcription factor largely generate only a single cell type *in vivo*. Rather few (about 16.7% in the murine cerebral cortex, see Gao et al., 2014) generate neurons and one type of glia and so far, to our knowledge, no stem cell‐generating neurons, astrocytes, and oligodendrocytes *in vivo* have been observed in the vertebrate CNS (Fig. 1).

*In vitro*, however, upon exposure to growth factors, at least some NECs generate both neurons and glial cells, for example neurons and astrocytes, or neurons and oligodendrocytes (Fig. 1; see, e.g., Qian et al., 2000; Williams et al., 1991; for review, see: Götz, 2013; Götz and Huttner, 2005). When NECs are exposed to neurosphere culture conditions, some (e.g., bearing CD44, Pollard et al., 2008) can generate all three types of cells, astrocytes, oligodendrocytes, and neurons, supposedly due to the upregulation of gliogenic transcription factors mediated by epidermal growth factor (EGF) and sonic hedgehog (SHH) signaling pathways (Gabay et al., 2003; Hack et al., 2004). Thus, important definitions to introduce here refer to the distinction between LINEAGE which is what a single cell does *in vivo* and POTENTIAL which is what a single cell CAN DO when exposed to a different environment either by transplantation or in culture (Fig. 1). Taken together, most NECs are unilineage and few bilineage *in vivo*, but some can exhibit multipotency *in vitro*.

Moreover, most NECs self‐renew for only a few cell divisions *in vivo* and in almost all brain regions they are fast replaced by the radial glial cells (RGCs) (Götz and Huttner, 2005; Sahara and O'Leary, 2009), such that RGCs are responsible for most of neurogenesis in most brain regions (for recent review, see De Juan Romero and Borrell, 2015; Taverna et al., 2014). One exception is the spinal cord where RGCs only appear at the end of neurogenesis and onset of gliogenesis (Barry and McDermott, 2005; Guérout et al., 2014; McDermott et al., 2005; Rowitch and Kriegstein, 2010). Thus, the earliest NSCs, the first progenitors in the developing CNS have only limited self‐renewal and are largely specified to generate a single or rather limited range of progeny *in vivo*. However, a subset of NECs can acquire multipotency and long‐term self‐renewal *in vitro* (Gabay et al., 2003; Hack et al., 2004; Pollard et al., 2006, 2008). Interestingly, specific signaling pathways, such as BMP, can even arrest these cells temporarily in quiescence (Martynoga et al., 2013), even though few if any of the NECs are quiescent *in vivo* (see, e.g., Furutachi et al., 2015; Hartfuss et al., 2001). Thus, the earliest NSCs are short‐lived and mostly unilineage but some can become multipotent and long term self‐renewing *in vitro*.

RGCs differ from NECs by expression of various genes and proteins later persisting in astrocytes, such as the glutamate transporters GLAST and Glt‐1, Glutamine Synthase, and Aldh1L1, or present in reactive astrocytes (vimentin, nestin, BLBP, DSD1‐proteoglycan, and Tenascin‐C) and adult NSCs (Table 1, see also Götz, 2013; Götz and Huttner, 2005; Kriegstein and Alvarez‐Buylla, 2009; Sirko et al., 2010; Taverna et al., 2014; von Holst et al., 2006). They share the apico‐basal polarity [with the long radial process attached to the basement membrane and the junctional complexes delineating apical membrane domains at the ventricular surface where, e.g., the glycoprotein prominin 1 (CD133) is located] (Götz and Huttner, 2005; Taverna et al., 2014). Both NECs and RGCs possess junctional complexes that change, however, in their molecular composition during development (Götz and Huttner, 2005; Rousso et al., 2012; Taverna et al., 2014) until the final cell type lining the ventricle the ependymal cells have differentiated with yet a different, rather leaky, junctional composition (Bruni, 1998; Jiménez et al., 2014).

So are RGCs then NSCs? *In vivo* and in primary cultures *in vitro* most RGCs generate only a single type of progeny, most of them neurons, some glia only, and similar to the NECs around 16.7% generate both neurons and glia (Fig. 1; Gao et al., 2014; Grove et al., 1993; Malatesta et al., 2003, 2000). Similar to the NECs, trilineage is not observed for RGCs *in vivo*, but only as multipotency *in vitro* upon growth factor exposure, such as in neurosphere culture conditions. In regard to self‐renewal, RGCs typically divide asymmetrically for a number of rounds generating different neuronal subtypes sequentially (for recent review, see Greig et al., 2013; Lodato et al., 2015). The RGC potential to generate different neuronal subtypes is increasingly limited during development, such that late RGCs can no longer generate deep layer neurons of the cerebral cortex that are generated only early (Desai and McConnell, 2000; Frantz and McConnell, 1996; Leone et al., 2008). Thus, many if not most RGCs do not self‐renew, as later RGCs differ in their fate restriction from the earlier RGCs. Moreover, RGCs divide maximally eight to nine rounds during neurogenesis and later disappear in most brain regions at the end of neurogenesis, when gliogenesis starts. They disappear either by self‐consuming symmetric neurogenic divisions or by generating or turning into glial cells, such as astrocytes or ependymal cells (Jacquet et al., 2009; Noctor et al., 2004; Paez‐Gonzalez et al., 2011). Taken together, RGCs like NECs show limited self‐renewal and give rise largely to a single type of progeny *in vivo*, but can do more upon growth factor exposure *in vitro* (Fig. 1).

Importantly, however, they are clearly more “potent” in fate and division than neuronal progenitor cells (such as the basal progenitors in the developing forebrain, see Borrell and Götz, 2014; Pilz et al., 2013; Taverna et al., 2014) that often divide only once, or if they divide more often they do so symmetrically enlarging the number of a single neuronal subtype. Thus, in regard to asymmetric divisions and the generation of different daughter cells RGCs and NECs are clearly different from neuronal progenitors and thereby fulfill the hallmark of self‐renewal even though only for a few rounds of cell division *in vivo* (in rodents: 2–4 for NECs; 6–8 for RGCs, see, e.g., Gao et al., 2014). Moreover, neuronal progenitors cannot form neurospheres (see, e.g., Pinto et al., 2008, unpublished data; Pollard et al., 2008), the *in vitro* read‐out for NSC potential, and hence lack multipotency and capacity for long‐term self‐renewal.

### NSCs During Postnatal Development: Peak of Glial Cell Expansion and Limited Self‐Renewal

After birth, in most mammalian brain regions neurogenesis has come to an end (for exceptions see below), while gliogenesis now prevails (see also Molofsky and Deneen, 2015; Takebayashi and Ikenaka, 2015). As discussed above, the view that the same NSCs that generated neurons during embryonic development would then generate the glial cells could be refuted for the vast majority of cells by clonal analysis (Gao et al., 2014). Thus, glial progenitors largely, though not exclusively (Gao et al., 2014), derive from ancestors that were not engaged previously in neurogenesis (see also for spinal cord lineages: Battiste et al., 2007; Mukouyama et al., 2006; Wu et al., 2006).

The postnatal period is thus dominated by large amplification of the glial lineages. At this time a dense band of proliferating cells forms at or just below the future white matter (WM) position as subventricular zone (SVZ). Only very few regions located mostly in the telencephalon have already a visible SVZ at embryonic stages that comprises however neuronal progenitor cells (the basal progenitors) and is most visible in regions with a large neuronal output (see De Juan Romero and Borrell, 2015). At postnatal stages the SVZ contains largely glial progenitors as shown by retroviral labeling of single cells and their progeny, e.g., in the rodent cerebral cortex SVZ. Clonal progeny consisted either of larger dispersed sets of oligodendrocytes and their progenitors and astrocytes or smaller clusters typically of a single class of glial cells, e.g., only astrocytes or only oligodendrocytes (Levison and Goldman, 1993, 1997; Lin and Goldman, 2009; Suzuki and Goldman, 2003; Zerlin et al, 2004). Molecular follow‐up studies then revealed that higher levels of the transcription factor Olig2 inhibit astrocyte and favor oligodendrocyte fate in these lineages (Marshall et al., 2005). In addition, single lineage progenitors are also distributed throughout the parenchyma, such as astrocyte progenitors generating groups of astrocytes within the cerebral cortex gray matter (GM) (García‐Marqués and López‐Mascaraque, 2013; Ge et al., 2012; Zerlin et al., 2004), eventually causing the evenly spaced distribution of astrocytes. Interestingly, while no neuronal progeny was observed from progenitors in the cerebral cortex at postnatal stages, exposure to hypoxia during the postnatal days 3–11 activated the generation of Tbr1+ spiny pyramidal neurons apparently from local GFAP+ cells (Bi et al., 2011). In addition, the exposure to low oxygen *in vivo* or *in vitro* also elicits the formation of multipotent and long‐term self‐renewing neurospheres from some GFAP+ cells in the postnatal cerebral cortex (Bi et al., 2011), reminiscent of the injury response of reactive astrocytes at later stages (see below).

In case of NG2 progenitors, they can divide symmetrically and asymmetrically to generate oligodendrocytes as well as the evenly spaced network of NG2‐glia in the adult brain (Dimou and Gallo, 2015; Nishiyama et al., 2009). Again, these cells are unilineage or bilineage *in vivo* (see, e.g., Zhu et al., 2008, 2011), and also remain in their lineage in primary culture. Under neurosphere culture conditions, i.e., in the presence of EGF and fibroblast growth factor 2 (FGF2) subsets of these glial progenitors also reveal multipotency (Dimou and Gallo, 2015; Kondo and Raff, 2000; Suslov et al., 2002).

In some brain regions, however, neurogenesis continues, such as the dentate gyrus (DG) that forms predominantly at postnatal stages and along the lateral wall of the lateral ventricle. During embryonic development, the latter region is called ganglionic eminence (GE) (see also below and Fig. 3) and generates the projection neurons of the basal ganglia as well as virtually all interneurons of the entire telencephalon, including the olfactory bulb (OB) (Anderson et al., 2002; Sultan et al., 2013), at postnatal stages only OB interneurons continue to be generated (with interesting subtype changes to adulthood: Weinandy et al., 2011). Accordingly, viral vector or genetic fate‐mapping‐based lineage tracing of cells in the postnatal SVZ revealed also neuronal progeny migrating to and settling in the OB (Luskin, 1993; Marshall et al., 2003; Zerlin et al., 2004). The cells at the origin of this lineage are GFAP+ (Ganat et al., 2006) and derive from neonatal RGCs (Merkle et al, 2004). However, GFAP‐CreERT2 (no superscript for ERT2)‐based fate mapping also revealed a surprising postnatal lineage of GABAergic neurons in the cerebral cortex (Ganat et al., 2006). As this lineage is no longer observed in adulthood, it will be interesting to determine to which extent these GABAergic neurons derive from the same progenitors as during embryonic development, namely the lateral wall of the lateral ventricle (Anderson et al., 2002; Southwell et al., 2014; Sultan et al., 2013), or from other regions such as the posterior periventricular region in the cerebral cortex (Caputi et al., 2013; Inta et al., 2008; Le Magueresse et al., 2011; Nakatomi et al., 2002). Importantly, however, no clonal analysis of these cells has yet been performed, such that it is unknown whether a single cell would give rise to different types of neural progeny *in vivo*.

### Adult NSCs: Limited Lineage and Self‐Renewal

The tissue‐maturation process during early postnatal life results in the gradual shrinkage of the VZ and SVZ accompanied by ependymal cell maturation, which leads to the formation of highly specialized area—the subependymal zone (SEZ) at the lateral wall of the lateral ventricles and the secondary neurogenic zone, the subgranular zone (SGZ) of the DG in the hippocampal formation (Kazanis et al., 2008; Kriegstein and Alvarez‐Buylla, 2009; Riquelme et al., 2008). Both these areas continuously contribute new neurons to the OB and the granule cell layer of the DG, respectively (Kriegstein and Alvarez‐Buylla, 2009) and retain some features from embryonic neurogenesis, often referred to as stem cell niches (see, e.g., Curtis et al., 2007; Kazanis et al., 2008; Riquelme et al., 2008).

So if the broader NSC criteria are only partially met during development, maybe the “real” NSCs appear only in adulthood as is the case, for example, in the hematopoietic system. The embryonic HSCs are dedicated to generate blood cells very fast and show limited self‐renewal, while adult HSCs appear late in embryogenesis and can self‐renew for about 15 life spans (one single HSC can reconstitute the hematopoietic system in serial transplants into 15 recipient mice sequentially, for review, see, e.g., Clapes and Robin, 2012). Adult NSCs were discovered by their multipotency *in vitro*, when cells from a thin area beneath the ependymal cell layer, the SEZ underlying the striatum were found to proliferate in medium containing EGF or FGF2 (Reynolds and Weiss, 1992; Richards et al., 1992) and likewise cells from the adult DG, OB, and hypothalamus could form multipotent and self‐renewing neurospheres (Gage et al., 1998, 1995; Robins et al., 2013; Vicario‐Abejón et al., 2003; Fig. 1). These cells can self‐renew for at least 10–20 passages and generate neurons, astrocytes, and oligodendrocytes upon differentiation *in vitro*—but do they do this also *in vivo*? To answer this question the progeny of a single cell must be followed *in vivo*. Clonal analysis can be done *in vitro* in the absence of growth factors observing the divisions and progeny of a single adult NSC by continuous single‐cell live imaging (Costa et al., 2011; Ortega et al., 2013). This revealed that without the addition of growth factors, adult NSCs isolated from the SEZ generate neurons only, via a series of amplifying progenitor divisions, but do not generate glia (Costa et al., 2011). Interestingly, as soon as EGF or FGF2 are added the NSCs revert to proliferation and gliogenesis (Costa et al., 2011). Thus, while *in vivo* most NSCs generate neurons only (Fig. 1), their multipotency *in vitro* is elicited by growth factors instructing gliogenesis (Fig. 1; Costa et al., 2011; Ortega et al., 2013).

Similarly, genetic fate mapping of murine adult NSCs *in vivo* suggests that single adult NSCs generate only neurons *in vivo* (for lineage in other vertebrates see Than‐Trong and Bally‐Cuif, 2015). By inducing genetic recombination in a small number of NSCs *in vivo* and then monitoring their genetically labeled progeny (Bonaguidi et al., 2011; Calzolari et al., 2015; Encinas et al., 2011), clonal analysis showed a rather limited self‐renewal of many (dentate gyrus) or most (SEZ) NSCs differentiating after two to four rounds of division. Like the RGCs (to which adult NSCs resemble closely (Fig. 1), see Beckervordersandforth et al., 2010; Calzolari et al.,^2015^; Kriegstein and Alvarez‐Buylla, 2009), the NSCs located in either the DG or the SEZ generate largely a single cell type. Notably, DG neurons are rather homogeneous while OB interneurons generated in the adult comprise diverse subsets (Lledo et al., 2008). Interestingly, however, distinct sets of OB interneurons are generated by distinct sets of NSCs located at distinct positions lining the lateral ventricle or even within the OB (Brill et al., 2009; Ihrie et al., 2011; Merkle et al., 2014, 2007; Vergaño‐Vera et al., 2014, 2009). Thus, it appears that the role models of NSCs in the mammalian nervous system, both embryonic and adult NSCs, are largely unilineage and with—as far as it is known so far—little self‐renewal *in vivo*, but can be instructed to multipotency and long‐term self‐renewal upon growth factor exposure *in vitro*. This implies that even for endogenous NSCs the revelation of their stem cell hallmarks largely relies on their *in vitro* expanded multipotency and self‐renewal.

## NSCs Outside the Neurogenic Niches in the Adult Mammalian CNS?

The above considerations prompt the very simple question to which extent some other cells in the adult brain parenchyma would also show a larger potential when exposed to such favorable conditions *in vitro*. Indeed, soon after neurospheres could be grown from the adult SEZ and DG, several other CNS regions were probed for this capacity. Especially from rats, neurospheres can be grown from several brain regions and even the spinal cord (Grande et al., 2013; Ohori et al., 2006; Palmer et al., 1999). This seems to be rather different in the mouse where few if any neurospheres can be derived from other regions than the SEZ, DG, and the hypothalamus (see, e.g., Babu et al., 2007; Robins et al., 2013; Sirko et al., 2013). Moreover, most of the very few neurospheres that are obtained from murine brain parenchyma outside the classical neurogenic niches cannot be passaged and do not give rise to neurons (Barnabé‐Heider et al., 2010; Meletis et al., 2008; Sirko et al., 2013, 2009). However, NSCs with neurosphere‐forming capacity may be a rare event in the brain parenchyma so it is important to bear in mind that about two to five among 10,000 cells dissociated from the adult murine brain (e.g., cerebral cortex) can form at least short‐term (one to two passages) self‐renewing neurospheres of which some are multipotent (Grande et al., 2013; Sirko et al., 2013).

The origin of these rare neurosphere‐forming cells is not known. While some refer to marker‐negative neural progenitor cells (Grande et al., 2013), others have suggested that the neurosphere‐forming cells in the adult CNS parenchyma are derived from NG2+ glia (Shihabuddin et al., 2000). Indeed, the only proliferating cell type in the adult brain parenchyma are NG2‐glia (Simon et al., 2011), even though most of them divide very slowly (Psachoulia et al., 2009; Simon et al., 2011). Accordingly, retroviral vectors integrating only in the genome of dividing cells also label cells when injected into the adult rat cerebral cortex and some of these can generate neurospheres (Grande et al., 2013). However, genetic fate mapping in mice has so far not lent support to NG2‐glia forming self‐renewing multipotent neurospheres (Barnabé‐Heider et al., 2010; Buffo et al., 2008; Meletis et al., 2008; Sabelström et al., 2013; Sirko et al., 2013). Thus, few if any cells generating multipotent, long‐term self‐renewing neurospheres can be obtained from the adult mammalian CNS, with important species differences however. Similarly, adult neurogenesis differs profoundly between mammals in a species‐specific manner with, e.g., apparent neuronal turnover and neurogenesis in humans (see, e.g., Ernst et al., 2014; Ninkovic and Götz, 2015).

### Reactive Astrocytes and Ependymal Cells with NSC Hallmarks After Injury

The situation changes when the parenchyma is exposed to injury. Several labs have reported a significant increase of neurosphere formation from the CNS parenchyma after injury—especially traumatic (TBI) or ischemic brain injury. The first report (Buffo et al., 2008) used genetic fate mapping of astrocytes to follow them through their behavior after stab wound injury in the adult mouse cerebral cortex—initially in still images (Buffo et al., 2008) and eventually by live *in vivo* imaging (Bardehle et al., 2013). Stab wound injury is a model of TBI with pronounced reactive gliosis (Norton et al., 1992; Pekny and Pekna, 2014; Robel et al., 2011). Astrocytes divide rarely if at all in the healthy brain, while proliferating cells with astrocyte morphology and markers (GFAP, GLAST, Aldh1L1, Glutamine synthase, and S100β) have been observed after injury (Amat et al., 1996; Buffo et al., 2008; Simon et al., 2011; Sirko et al., 2009). However, it had been proposed that these proliferating cells with astrocyte markers may derive from glial progenitors, the NG2‐glia population that divide already in the healthy brain (Dimou and Götz, 2014; Reynolds and Hardy, 1997; Simon et al., 2011). Conversely, mature protoplasmic astrocytes were considered as permanently postmitotic cells, similar to mature neurons and mature oligodendrocytes. Permanently postmitotic cells, such as neurons and oligodendrocytes, cannot resume cell division even after injury, and rather undergo polyploidy or cell death when cell cycle genes are activated (Arendt, 2012). If this were similar for astrocytes, proliferating reactive GFAP+ cells may rather be derived from normally proliferative NG2‐glia.

Genetic fate mapping could resolve this important issue by labeling the respective glia population prior to injury (turning on a marker gene, such as β‐galactosidase or GFP using GLAST^CreERT2^ knock‐in or GFAP‐CreERTm mice to label astrocytes or Olig2CreERTm mice to label NG2‐glia after tamoxifen addition; Buffo et al., 2008; Dimou et al., 2008; Shimada et al., 2012) and then following the labeled cells during and after injury. Note that all of these Cre lines also label endogenous NSCs in SEZ and hippocampal SGZ, such that the injury must be performed at reliable distance from these sites to allow conclusion about parenchymal astrocytes (for such controls, see Buffo et al., 2008). This is of particular relevance for WM injuries, as SEZ progeny are more prone to be recruited there (Benner et al., 2013; Etxeberria et al., 2010).

Parenchymal GM astrocytes that were nonproliferative prior to injury indeed reactivated proliferation as detected by 5‐bromo‐2′‐deoxyuridine (BrdU) incorporation or Ki67 immunostaining within the first week after stab wound injury (Buffo et al., 2008; Shimada et al., 2012; Sirko et al., 2013). Importantly, this could be further confirmed by live *in vivo* imaging unequivocally identifying parenchymal protoplasmic astrocytes that divided and generated two bushy protoplasmic astrocytes typically remaining in close vicinity to each other (Bardehle et al., 2013). Proliferating astrocytes are a subset of reactive astrocytes that only ever divide once and are enriched at juxtavascular positions (Bardehle et al., 2013; for review, see Dimou and Götz, 2014; Götz and Sirko, 2013). Thus, astrocyte proliferation can be reactivated, but only in a subset of astrocytes ranging between 15 and 40% of all astrocytes. Importantly, however, despite activation of proliferation and expression of some more immature markers (such as nestin and DSD1, see Table 1), murine cerebral cortex GM astrocytes stay within their lineage (Fig. 1) and no other progeny was observed neither by genetic fate mapping nor by live imaging (Bardehle et al., 2013; Buffo et al., 2008; Shimada et al., 2012; Sirko et al., 2013). Indeed, the concept that astrocytes can resume proliferation after injury but remain within their lineage has also been corroborated in the far distant region of the spinal cord (Barnabé‐Heider et al., 2010; Grégoire et al., 2015). These new insights into a subset of reactive astrocytes resuming proliferation have important consequences in regard to their function after brain injury (Dimou and Götz, 2014). As proliferation is the only way to increase astrocyte numbers after injury—given the absence of migration (Bardehle et al., 2013)—and this occurs at the juxtavascular interface, a key role of astrocytes may occur at the blood vessel interface, e.g., in restricting immune cell invasion (Dimou and Götz, 2014; Sofroniew, 2014).

However, like for endogenous NSCs, the lineage restriction observed *in vivo* may be due to the local environment and the potential of reactive astrocytes may be larger *in vitro*. Notably, the adult brain parenchyma is very gliogenic and not supportive of neurogenesis. For example, SEZ‐derived neurosphere cells or SEZ‐derived neuroblasts that normally readily generate neurons are reverted to gliogenesis when transplanted into the adult injured brain parenchyma (e.g., Seidenfaden et al., 2006; Shihabuddin et al., 2000; for review, see Dimou and Götz, 2014). To explore the potential of reactive astrocytes genetically fate‐mapped astrocytes were exposed to the standard neurosphere culture conditions containing EGF and FGF2 at 3–5 days postinjury to allow NSC proliferation and self‐renewal. Indeed, neurosphere formation was much higher (5–10 times) after traumatic or ischemic brain injury (Buffo et al., 2008; Sirko et al., 2013, 2009) and genetically labeled astrocytes exhibited NSC properties of self‐renewal (passages for more than five times) and multipotency as typically one‐third of the neurospheres generated all three cell types, neurons (firing action potential), oligodendrocytes, and astrocytes (Buffo et al., 2008; Sirko et al., 2013). Genetic fate mapping revealed that most of the neurospheres were derived from genetically (Glast^CreERT2^/GFP) labeled astrocytes and isolation of labeled reactive astrocytes by FACS showed that up to 1 in 18 viable GFP+ cells could form a neurosphere (Buffo et al., 2008). Thus, about 5% of all reactive astrocytes have NSC properties revealed *in vitro*.

Interestingly, the source of cells with NSC potential is different in the spinal cord, where astrocytes fate‐mapped in Connexin30‐CreERT2 mouse lines formed virtually no neurospheres and hence revealed little to no multipotency, while ependymal cells did (Barnabé‐Heider et al., 2010; Meletis et al., 2008). Thus, different glial subtypes may retain NSC/neurosphere‐forming potential in different CNS regions.

But which are the functional similarities or differences of reactive astrocyte‐derived NSCs and the “true” NSCs that contribute to neurogenesis in the adult brain? In other words, are reactive astrocytes or ependymal cells really “true” NSCs? In regard to self‐renewal, there is a quantitative difference between reactive astrocytes dividing only one time and NSCs about three to four times *in vivo* (Bardehle et al., 2013; Bonaguidi et al., 2011; Calzolari et al., 2015; Encinas et al., 2011). *In vitro*, however, both populations can self‐renew for more rounds of cell divisions, supposedly triggered by the amounts of growth factors. Thus, NSCs and reactive astrocytes can self‐renew for a number of times, but do so in a more limited number *in vivo*. The same conclusion is reached for multipotency. *In vivo*, endogenous NSCs and reactive astrocytes generate one set of progeny: neurons in the case of endogenous NSCs in the SEZ or DG, astrocytes in the case of the hypothalamic NSCs (Robins et al., 2013; for earlier stages, see Haan et al., 2013) or reactive astrocytes (Bardehle et al., 2013; Buffo et al., 2008; Sirko et al., 2013). Interestingly, reactive ependymal cells in the spinal cord generate also astrocytes migrating from the ventricular lining to the injury site (Barnabé‐Heider et al., 2010; Meletis et al., 2008; Sabelström et al., 2013). Thus, *in vivo* all of these NSCs, both the endogenous NSCs and the reactive glia, are either unilineage or bilineage, but never generate neurons and glia. Their potential, however, is clearly much larger *in vitro* (Fig. 1) where reactive astrocytes and ependymal cells generate neurons and the two main types of glial cells (Barnabé‐Heider et al., 2010; Buffo et al., 2008; Meletis et al., 2008; Sabelström et al., 2013; Shimada et al., 2012; Sirko et al., 2013). Thus, like endogenous NSCs from the developing and adult brain, also reactive astrocytes and ependymal cells have limited lineage and self‐renewal *in vivo*, but comply with the stem cell criteria of longer self‐renewal and multipotency *in vitro*.

### Probing Potential by Transplantation

At this point it is also important to mention that neurons generated by neurospheres are poorly defined *in vitro*, such that the full neuronal maturation into a specific subtype remains yet to be determined. As neuronal subtype maturation may be impaired due to altered signaling (this is the case for SEZ NSCs, see, e.g., Brill et al., 2009) or simply difficult to detect *in vitro*, the ideal test for the potential of NSCs is transplantation. As mentioned above, neurospheres derived from endogenous adult NSCs largely fail to generate neurons when transplanted outside the neurogenic niches, i.e., most part of the adult brain parenchyma. However, when transplanted into their own neurogenic sites, they generate neurons apparently corresponding to the respective neuronal subtype even after exposure to the EGF and FGF2 signals *in vitro* (Codega et al., 2014; Merkle et al., 2007; Shimada et al., 2012). To which extent neurospheres derived from one site (e.g., the DG) can generate neurons of another site (e.g., the OB)—as suggested some while ago (Suhonen et al., 1996) remains to be examined in more detail with modern techniques controlling for cell fusion (Brilli et al., 2013). More recently, SEZ‐derived neurosphere cells have been shown to differentiate into DG neurons only when expressing the correct neurogenic transcription factor fate determinants (Chen et al., 2012). The most striking example upon wide‐spread multilineage contribution of adult SEZ‐derived neurospheres was observed after transplantation into the embryonic brain apparently contributing to neurons in many different regions (Neumeister et al., 2009). However, the neuronal progeny was not examined in detail in this study, especially not in regard to their function and connectivity. Thus, it remains largely unknown to which extent even the normal endogenous adult NSCs can contribute to neuronal subtypes of other regions. Moreover, many of these studies did not address the issue of cell fusion, which is rather prevalent for NSCs after *in vitro* culture (Brilli et al., 2013).

Reactive astrocyte‐derived neurospheres have been transplanted into the SEZ and while they proliferate no neuronal progeny was observed (Shimada et al., 2012). This clearly suggests that these cells cannot react to the neurogenic stimuli present in the neurogenic SEZ. This may be due to the distinct regional origin as the adult SEZ derives largely from the lateral GE as mentioned above (Young et al., 2007). Indeed, cells derived from the cerebral cortex never generate OB interneurons during development and may hence not be able to respond to these cues. Thus, it will be an important test to examine the progeny of reactive astrocytes in a niche generating the appropriate subtype of neurons, i.e., the embryonic cerebral cortex. Taken together, reactive astrocyte‐derived neurosphere cells clearly exhibit multipotency *in vitro*, but their neurogenic potential remains to be further explored and may well require additional signals as these cells are derived from a cell type that has stopped to generate neurons some while ago. Indeed, neurospheres derived from NSCs undergoing neurogenesis *in vivo* generate neurons to a lower proportion than *in vivo* (due to the gliogenic signals, see below), but each neurosphere generates neurons. Conversely, only one‐third of all neurospheres derived from reactive astrocytes generate neurons (Sirko et al., 2013; Fig. 1), suggesting that also *in vitro* their neurogenic potential is lower.

This may imply that injury conditions *in vivo* and neurosphere culture conditions *in vitro* may only activate part of the neurogenic capacity in the subset of reactive astrocytes. Taken together, a few astrocytes can activate NSC hallmarks after injury and these properties do not differ much from the behavior of endogenous adult NSCs in regard to long‐term self‐renewal and multipotency *in vitro* or their limited progeny and self‐renewal *in vivo*. However, the key difference is in regard to their default lineage *in vivo*, which is neurogenic for the endogenous NSCs and gliogenic for the NSCs emerging from reactive glia.

## Factors Regulating Reactive Astrocyte Multipotency and Neuronal Progeny

The crucial issue emerging from the above is thus to identify the signals that retain reactive astrocytes in their lineage *in vivo* with the final aim to relieve them from this restriction. Notch signaling is a prime candidate as its levels determine already in RGCs during neurogenesis whether they undergo neurogenesis or gliogenesis. High sustained levels of Notch retain cells as RGCs and interfere with neurogenesis, while oscillating Notch signaling interrupted by high Ngn and low Hes levels allow RGCs and NSCs to generate neurons (Imayoshi and Kageyama, 2014; Shimojo et al., 2011). Notch signaling is critical for NSC properties of reactive astrocytes in neurosphere cultures (Shimada et al., 2012), consistent with the role of Notch in NSC self‐renewal (see Ninkovic and Götz, 2014; Than‐Trong and Bally‐Cuif, 2015). Excitingly, recent work from the Frisén lab has shown that striatal astrocytes are particularly dependent on this signaling pathway as deletion of the key mediator of Notch signaling, Rbpjκ, is sufficient to elicit neurogenesis (doublecortin and NeuN+ cells) from striatal but not other astrocytes (Magnusson et al., 2014). Interestingly, striatal astrocytes also generate some immature neurons upon injury conditions (stroke) that elicit a reduction in Notch signaling (Magnusson et al., 2014), as do ependymal cells underlying the striatum (Carlén et al., 2009). Striatal glial cells may therefore appear to be particularly easy to activate toward neurogenesis, a possible remnant from a hidden neurogenic program present in rabbits and humans (Ernst et al., 2014; Luzzati et al., 2006). Thus, these data highlight the region‐specific diversity of astrocytes (see also Molofsky and Deneen, 2015) as astrocytes in most other brain regions do not generate neurons after injury (Barnabé‐Heider et al., 2010; Buffo et al., 2008; Magnusson et al., 2014; Sirko et al., 2013) and cannot be instructed to do so by deleting Rbpjκ (Magnusson et al., 2014). However, other signals may restrict astrocytes from converting to neuronal lineage in other regions.

In regard to the NSC properties of reactive astrocytes in the cerebral cortex, SHH turned out to be a key signaling mediator. When the SHH signaling mediator smoothened is deleted in adult astrocytes, proliferation of reactive astrocytes after traumatic injury is much reduced and so is their neurosphere formation (Sirko et al., 2013). SHH levels are highest in the blood plasma and cerebrospinal fluid (CSF) which both gain access to the brain parenchyma after invasive brain injury. This explains why reactive astrocytes do not resume proliferation in noninvasive injury, conditions such as amyloidosis or induced cell death of neurons (Sirko et al., 2013). Importantly, SHH levels appear not to be saturating as the smoothened agonist (SAG) can further increase proliferation and neurosphere formation of reactive astrocytes and even their neurogenic capacity (Sirko et al., 2013), but even elevated SHH signaling *in vivo* is not sufficient to promote neurogenesis from reactive astrocytes *in vivo* (Sirko et al., 2013 and Frik et al., unpublished data). Surprisingly, one of the two factors contained in the neurosphere medium, the FGF2, rather inhibits the proliferation and reactive nature of astrocytes *in vivo* (Kang et al., 2014). Again, this effect was specific to the cerebral cortex and different in other regions. These data thus further highlight the importance to unravel the signaling network in the region‐specific environment *in vivo*.

One aspect of understanding the signaling pathways elicited by injury is to alleviate scar formation. Indeed, some of these signaling pathways seem to affect scar formation (for details, see Grégoire et al., 2015). This is the case for FGF signaling (Kang et al., 2014) or the intermediate filaments GFAP and vimentin whose deletion potently reduced scar formation (Pekny and Pekna, 2014; Pekny et al., 1999). Interestingly, intermediate filaments also act by targeting membrane proteins to the cell surface and may hence play a role also in FGFR‐mediated signaling (Kang et al., 2014). Most importantly, the signals affecting NSC or even neurogenesis potential of reactive astrocytes and the signals that lessen scar formation may be closely linked. Transplantation of immature glial cells into injury sites has often yielded beneficial effects (Shear et al., 2004), suggesting that the factors released by various transplanted stem cells, including mesenchymal stem cells, alleviate scar formation and neuronal death in so‐called by‐stander effects. This implies that the factors released by NSCs or glial progenitors are beneficial. Indeed, recruitment of NSCs from endogenous sources appears to have the same effects. This is best illustrated by reactive astrocytes recruited from NSCs in some brain regions. For example, when injury stretching into the WM is placed into rostral areas of the mouse cerebral cortex, astrocytes from the SEZ are recruited to the injury site in a thrombospondin 4‐dependent manner exerting beneficial effects (Benner et al., 2013). Similarly, ependymal cells in the spinal cord are activated by traumatic injury and generate diverse types of glial cells, including GFAP+ cells migrating to the injury site and releasing neuroprotective factors (Sabelström et al., 2013; for further details, see Grégoire et al., 2015). A similar process is taking place also in the DG where neurogenic NSCs are recruited to generate reactive astrocytes at the expense of neurogenesis upon injury (Encinas et al., 2011). These data highlight the dark side of activating NSCs toward the generation of reactive astrocytes as this depletes NSCs and negatively affects later neurogenesis in this region (see, however, nonmammalian vertebrates as reviewed in Than‐Trong and Bally‐Cuif, 2015), while apparently improving scar formation or neuronal survival at the site of injury.

Taken together, there are different cellular sources contributing to reactive astrocytes in specific brain regions and distinct signals regulate reactive astrocyte proliferation and NSC properties in different brain regions. These considerations thus bring us back to the more general questions of regional differences between endogenous NSCs and to which extent reactive astrocytes (or ependymal cells) may resemble endogenous NSCs from the same or different regions.

## Genome‐Wide Expression Analysis of Embryonic and Adult NSCs and Protoplasmic and Reactive Astrocytes

To answer these questions more globally and in an unbiased manner, genome‐wide expression analysis should be well suited. While the above review shows key similarities between NSCs and reactive astrocytes in terms of some marker genes (Table 1) and functional aspects (Table 2), these reflect only a minute part of the overall gene expression and hence functional plasticity of these cells.

**Table 1:** Similarities and Differences Between Glial Cell Types in Terms of Some Marker Genes Expression

Protein	Neuroepithelial cells	Radial glia early	Radial glia late	Adult neural stem cell	Mature astroglia	Reactive astroglia	Ependymal cell (lateral ventricle)
GFAP	−	−/+	+/++	+++	−/++	+++	+
GLAST (Slc1a3)	−	++	++	++	+++	+++	++
GLT1 (Slc1a2)	−	−	+	++	+++	++	++
Glutamine synthetase	−	−	+	++	+++	+++	−
S100‐β	−	−	+	+	++	+++	+++
Connexin 43 (Gja1)	−	−	++	+++	+++	+++	++
Aquaporin 4	ND	ND	ND	++	+++	+++	+
KIR 4.1/2.1	−	+	++	+++	+++	++	++
Aldlhl1	−	−	+	+	+++	+++	++
Nestin (RC1/RC2)	+++	+++	+++	+++	−	+++	++
Vimentin	−	+	++	+++	−	+++	+++
BLBP	++	+++	+++	+++	−	+++	−
TN‐C	−	+++	+++	++	−	+++	−
Phosphacan/DSD‐1	−	+++	+++	+++	−	++	−
Sox2	+++	+++	+++	+++	++	+++	++

Note that cell heterogeneity is not incorporated in this table. Based on Götz (2013).

**Table 2:** Functional Properties Shared by Astrocytes, Ependymal Cells, Radial Glia, and Adult Neural Stem Cells

Protein	Neuroepithelial cells	Radial glia early	Radial glia late	Adult neural stem cell	Mature astroglia	Reactive astroglia	Ependymal cell (lateral ventricle)
Glutamate uptake	−	+	++	+++	+++	+++	++
K‐conductance at rest	−	−	++	++	+++	++	++
Glycogen storage	−	+	++	++	+++	+++	+++
Gap‐junctions/hemichannels/Ca‐waves	ND	+++	+++	+++	+++	++	++
Blood vessel contact/blood flow regulation	−	+	++	+++	+++	+++	−
Cell division	+++	+++	++	++	−	++	−
Multipotency	+++	+++	++	+++	−	+	−
Self‐renewal	++	++	+	+++	−	+	−

Based on Götz (2013).

The limitation of the few “marker genes” examined so far becomes immediately obvious when alleged “NSC markers,” such as nestin, Sox2, DSD1, and BLBP, are examined in reactive astrocytes (Table 1), where they are widespread, even in injury conditions when reactive astrocytes do not acquire proliferation and neurosphere‐forming potential (Sirko et al., 2013). Thus, these few proteins are not suitable for delineating NSCs from other glial cells. Rather, genome‐wide expression analysis is required to delineate parenchymal astrocytes from cells with NSC properties and unravel how reactive astrocytes fit into these comparisons.

Such genome‐wide expression analysis could not only shed some light onto global similarities between these cell types but also help to understand which of these cells are more similar to each other. For example, one may expect that adult NSCs and embryonic NSCs may be most similar given their neurogenic progeny, localization at the ventricle, epithelial hallmarks (for recent review, see: Götz, 2013; Taverna et al., 2014), and NSC properties. On the other hand, cells isolated from the adult brain may be globally more similar to each other, and we know that adult NSCs also differ in many aspects from embryonic NSCs/RGCs, such as their speed of proliferation and their junctional properties.

**Figure 2:**
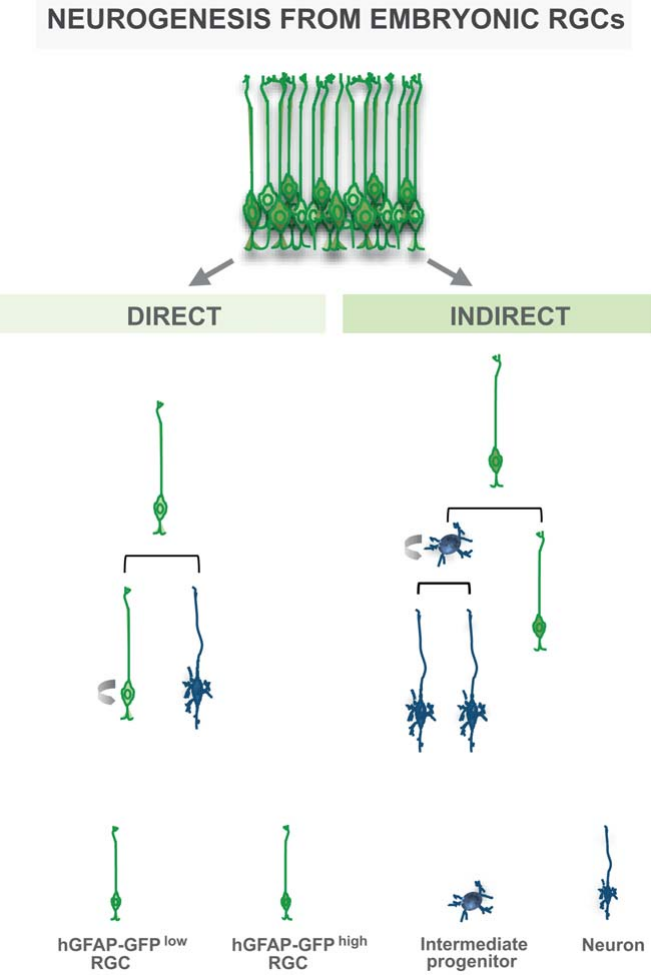
The lineage heterogeneity of radial glial cells from the cerebral cortex of embryonic day 14 mice. Radial glial cells comprise different sets of progenitors, relating to differences in gene expression and neurogenic capacity. Indirectly neurogenic RGCs generate intermediate basal progenitors and had higher levels of hGFAP‐GFP (hGFAP‐GFP^high^), while RGCs that give rise directly to neurons had lower levels of GFP (hGFAP‐GFP^low^). These subtypes of RGCs can be separated from the cerebral cortex at E14 on the basis of the level of GFAP‐driven GFP and the different modes of neurogenesis from radial glial cells were revealed by live imaging (for review, see Götz, 2013).

**Figure 3:**
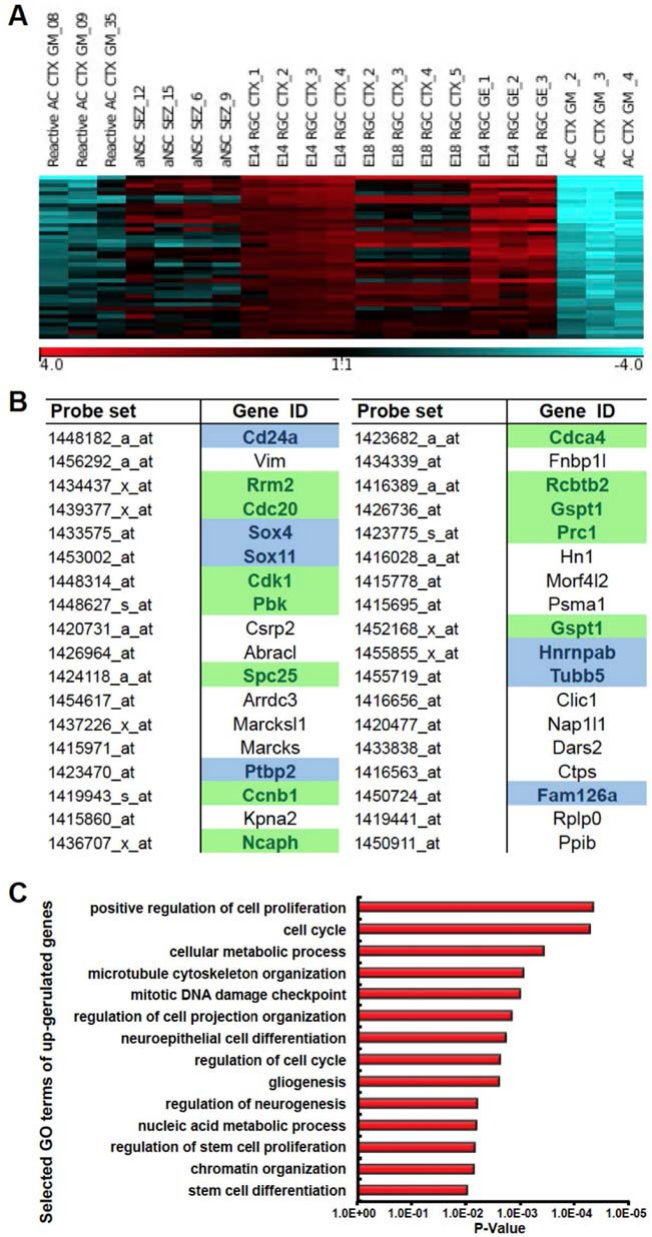
The comparative genome‐wide analysis of different astroglial cell sets from the embryonic and adult mouse forebrain. Genes significantly enriched (more than fivefold) in reactive astrocytes (Reactive AC CTX GM), adult NSCs from the subependymal zone (aNSC SEZ), and RGCs from different stages and regions of the telencephalon (E14 RGC CTX, E18 RGC CTX, and E14 RGC GE) in comparison to protoplasmic astrocytes (AC CTX GM) are plotted as a heat map to illustrate similarity in gene expression between astrocytes sorted from the injured adult mouse cerebral cortex at the peak of their proliferative activity and RGCs/NSCs (the normalized values are plotted on a log_2_ color scale, with blue representing low expression and red representing high expression) (**A**). Thirty‐six candidate genes derived from (A) whose expression differs significantly between cells with stem cell or progenitor phenotype and mature astrocytes (**B**). (**C**) Bars show the significantly enriched GO terms associated with the candidate genes listed in B. AC, astrocyte; CTX, cortex; E, embryonic day; GE, ganglionic eminence; GM, gray matter; NSC, neural stem cell.

Here, we compare data obtained in the same lab by the same isolation technique. Fluorescence‐activated cell sorting (FACS) was used to isolate RGCs from the developing cerebral cortex (dorsal telencephalon) both at mid‐neurogenesis (embryonic day, E, 14) and at the end of neurogenesis (E18) (Pinto et al., 2008), as well as adult NSCs and astrocytes from the forebrain (Beckervordersandforth et al., 2010). To selectively isolate embryonic or adult NSCs we used a dual marker strategy. Antibodies directed against the apical membrane protein prominin 1 allowed selectively isolating cells by FACS that have an apical membrane domain at the ventricle where prominin 1 is enriched. This is the case exclusively for RGCs during embryogenesis, which were in addition labeled by hGFAP‐driven GFP (Pinto et al., 2008). As GFP is also inherited to the progeny generated by NSCs the dual labeling is essential to separate NSCs (that are prominin 1+ and hGFAP‐eGFP+) from their progeny, which are only GFP+ (Pinto et al., 2008). This screen therefore allowed separating different sets of RGCs based on their hGFAP‐eGFP expression level in combination with prominin 1 from E14 cerebral cortex. RGCs that generate intermediate basal progenitors had higher levels of hGFAP‐eGFP (E14Cortex GFP high in Fig. 2; E14 RGC CTX in Fig. 3A), while RGCs that give rise to only few of these amplifying progenitors, but rather self‐renew, had lower levels of GFP (Fig. 2; Pinto et al., 2008). This yielded the identification of key transcriptional regulators of these amplifying progenitors (AP2γ promoting amplifying progenitors and Trnp1 inhibiting it; Pinto et al., 2009; Stahl et al., 2013). Notably, these differences no longer exist in E18 cerebral cortex RGCs, when RGCs generate largely glial progeny (Pinto et al., 2008; E18 RGC CTX in Fig. 3A). As most of the adult NSCs located in the adult SEZ are derived from the ventral telencephalon, the GE, we also isolated RGCs from this region at mid‐neurogenesis (E14 RGC GE; Falk, Pinto, Irmler, Götz, unpublished data).

For isolation of adult NSCs we used the same dual labeling approach with an antibody against the apical membrane protein prominin 1 and hGFAP‐eGFP to detect astroglial/radial glial‐like cells with an apical process extending to the lateral ventricle (Beckervordersandforth et al., 2010). This was particularly critical for the adult SEZ, as hGFAP‐eGFP not only persists in the progeny of NSCs but also labels many surrounding astrocytes and prominin 1 is highly expressed by the multiciliated ependymal cells that also line the ventricle and need to be discriminated as prominin1+/hGFAP‐eGFP− (Beckervordersandforth et al., 2010; Fischer et al., 2011). Thus, only the dual labeling allowed comparing the gene expression profile of adult NSCs (quiescent and actively proliferating, for further discrimination of these adult NSC subtypes see Codega et al., 2014; Mich et al., 2014) to their ependymal cell neighbors and to parenchymal protoplasmic astrocytes from the same mouse line (hGFAP‐eGFP, Nolte et al., 2001) isolated from the diencephalon omitting the hypothalamic region (Beckervordersandforth et al., 2010). Our main aim of this study was to identify novel molecular regulators of adult neurogenesis—which are the genes highly expressed in adult NSCs and lowest in ependymal cells and parenchymal astrocytes (Beckervordersandforth et al., 2010).

For the discussion here it is however very important to highlight another conclusion of this work, namely that none of the *bonafide* astrocyte “markers,” not even the new Aldh1l1 (Cahoy et al., 2008), is specific for protoplasmic astrocytes. Rather all of the alleged astrocyte “markers,” including GLAST, GLT‐1, Glutamine synthase, and S100β, are expressed by adult NSCs, ependymal cells, and protoplasmic astrocytes at varying levels (Table 1). Accordingly, none of these markers can be used to reliably separate either of these populations. For example, the GLAST^CreERT2^ mouse line labels adult NSCs, as well as most parenchymal astrocytes and many ependymal cells, when induced to activate reporter gene expression (Ninkovic et al., 2007, 2013). To promote identification of astrocyte‐specific markers, we also included a list of genes that is highly expressed in diencephalic astrocytes, but low in NSCs and ependymal cells (Beckervordersandforth et al., 2010). Interestingly, however, isolating GM astrocytes from other brain regions, such as the cerebral cortex (AC CTX GM in Fig. 3A; Sirko et al., unpublished data) revealed some profound region‐specific differences between astrocytes from the diencephalon, supporting the recent concept of profound region‐specific differences between astrocytes (Hochstim et al., 2008; Molofsky et al., 2014; Tsai et al., 2012) also at the genome‐wide expression level. This raises the interesting concept that we may search in vain for a pan‐astrocyte marker, delineating these cells from NSCs. Either there are broad commonly expressed genes, but then they include gray and white matter astrocytes as well as NSCs, or there are too many differences between subsets of astrocytes to discover “markers” applicable for all of them. At least for forebrain astrocytes, however, now gene lists can be assembled comprising genes highly expressed in astrocytes isolated from the murine diencephalon and cerebral cortex by our lab (Beckervordersandforth et al., 2010; Sirko et al., unpublished) and the Barres lab (Cahoy et al., 2008; Zamanian et al., 2012) and low in adult NSCs, ependymal cells, neurons, oligodendrocytes, NG2‐glia, and in embryonic RGCs. To which extent these mRNA profiles prove to be useful at protein levels as new marker proteins and to which extent it is possible to find astrocytes, but not NSCs, across different regions expressing a gene in common remains to be seen.

Finally, there are several sets of genome‐wide expression data now for astrocytes sorted from the postnatal or adult mouse cerebral cortex without and with injury (Orre et al., 2014; Zamanian et al., 2012). As we used the same procedure (FACS sorting, two‐cycle RNA amplification) and same arrays (Affymetrix MOE430 2.0), we compare here our data on genome‐wide expression analysis of protoplasmic and reactive astrocytes at 5 days after stab wound injury and aNSCs (Fig. 3). In addition, data from RGCs (Pinto et al., 2008) generated on the same array type were included in the analysis. To at least partially correct for differences due to varying RNA amplification methods used for embryonic and adult samples an additional array dataset generated from the same sample by the two amplification methods was used. Thus, these data can serve as a basis for discussion, which is what we do in this review.

## Neural Stem Cell Similarities and Differences

Although all these genome‐wide expression data are from the same lab and the same arrays, batch effects may introduce artificial differences. Therefore, we focus here on similarities across all the different batches, and in particular, on the similarities in gene expression between embryonic NSCs, the RGCs, adult NSCs, and reactive astrocytes. Analysis of the genes expressed commonly higher in the reactive astrocytes and embryonic and adult NSCs compared with the mature cerebral cortex astrocytes (Fig. 3A) revealed many genes involved in regulating proliferation, which discriminates the NSCs and a subpopulation of reactive astrocytes from parenchymal protoplasmic astrocytes (Fig. 3B). Also typical reactive astrocyte genes, like vimentin, are higher in all NSCs and reactive astrocytes (Table 2). Interestingly, among the significantly enriched gene ontology terms in embryonic and adult NSCs and reactive astrocytes, but low in protoplasmic astrocytes some are related to neurogenesis, stem cell differentiation, or NEC differentiation (Fig. 3C). Indeed, some genes related to neurogenesis become re‐expressed in reactive astrocytes, such as the top gene *CD24*, *Sox4*, and *Sox11* (Ninkovic et al., 2013), and *Ptbp2* (Fig. 3B). Interestingly, however, the heatmap also reveals that these genes are expressed at much lower levels in the reactive astrocytes compared with embryonic or adult NSCs (Fig. 3A), even though still significantly higher than in protoplasmic adult cerebral cortex astrocytes (Fig. 3A). Taken together, this analysis reveals a significant set of commonly activated genes between endogenous NSCs and reactive astrocytes including those related to neurogenesis (Fig. 3).

This genome‐wide expression analysis can now serve as a starting point to rather focus on the neurogenesis genes that are not sufficiently activated in reactive astrocytes in comparison to endogenous NSCs. In this regard, it is also obvious that much of the neurogenic priming, i.e., elevated mRNA levels of neurogenic transcription factors, such as Pax6, Arx, and Dlx2, in endogenous NSCs (Beckervordersandforth et al., 2010) is missing in reactive astrocytes (with the exception of Sox4 and Sox11), consistent with their limited neurogenic capacity. However, reactive astrocytes that proliferate and have stem cell capacity are a subset ranging from about 20% that proliferate and maximally 5% that can form neurospheres. Thus, expression of NSC genes may be simply diluted by other reactive astrocytes. This brings us to the next level of analysis, namely aimed at subsets of astrocytes with specific properties. Indeed, astrocytes at juxtavascular positions, the population that is most proliferative after injury (Bardehle et al., 2013) is a subset with their own progenitors as recently demonstrated by clonal analysis (Martín‐López et al., 2013). This suggests that juxtavascular astrocytes may be a specific population of cerebral cortex astrocytes and isolation of this subset may then reveal to which extent these share even more similarities to the SEZ NSCs or rather are their own kind.

## Concluding Remarks

Thus, although the population analysis of NSCs isolated from the embryonic and adult telencephalon in comparison to protoplasmic astrocytes from the forebrain has provided us with key insights into molecular mechanisms regulating their distinct fates and highlighting the limitation of the present marker proteins as well as the genome‐wide expression differences, the next challenge is understanding the cellular heterogeneity. This will not only provide insights into heterogeneity of endogenous NSCs and teach us important lessons about their diversity but also reveal to which extent “common” hallmarks may exist at all. Similarly, single‐cell analysis (within their own technical limitations) of astrocytes will allow us determining their subtype identity and possibly predicting why some subtypes do not react even to a strong injury while others react by polarization and yet others by proliferation (Bardehle et al., 2013; Dimou and Götz, 2014). As ever, genome‐wide expression analysis is best used to understand functional differences and then tackle these at the functional level to elicit the desired phenotype, e.g., neurogenesis in case of brain injury.

## References

[glia22850-bib-0001] Amat JA , Ishiguro H , Nakamura K , Norton WT. 1996 Phenotypic diversity and kinetics of proliferating microglia and astrocytes following cortical stab wounds. Glia 16:368–382. 8721677 10.1002/(SICI)1098-1136(199604)16:4<368::AID-GLIA9>3.0.CO;2-W

[glia22850-bib-0002] Anderson SA , Kaznowski CE , Horn C , Rubenstein JL , McConnell SK. 2002 Distinct origins of neocortical projection neurons and interneurons in vivo. Cereb Cortex 12:702–709. 12050082 10.1093/cercor/12.7.702

[glia22850-bib-0003] Arendt T. 2012 Cell cycle activation and aneuploid neurons in Alzheimer's disease. Mol Neurobiol 46:125–135. 22528601 10.1007/s12035-012-8262-0

[glia22850-bib-0004] Babu H , Cheung G , Kettenmann H , Palmer TD , Kempermann G. 2007 Enriched monolayer precursor cell cultures from micro‐dissected adult mouse dentate gyrus yield functional granule cell‐like neurons. PLoS One 2:e388. 17460755 10.1371/journal.pone.0000388 PMC1849968

[glia22850-bib-0005] Bardehle S , Krüger M , Buggenthin F , Schwausch J , Ninkovic J , Clevers H , Snippert HJ , Theis FJ , Meyer‐Luehmann M , Bechmann I , Dimou L , Götz M. 2013 Live imaging of astrocyte responses to acute injury reveals selective juxtavascular proliferation. Nat Neurosci 16:580–586. 23542688 10.1038/nn.3371

[glia22850-bib-0006] Barnabé‐Heider F , Göritz C , Sabelström H , Takebayashi H , Pfrieger FW , Meletis K , Frisén J. 2010 Origin of new glial cells in intact and injured adult spinal cord. Cell Stem Cell 7:470–482. 20887953 10.1016/j.stem.2010.07.014

[glia22850-bib-0007] Barry DS , McDermott KW. 2005 Differentiation of radial glia from radial precursor cells and transformation into astrocytes in the developing rat spinal cord. Glia 50:187–197. 15682427 10.1002/glia.20166

[glia22850-bib-0008] Battiste J , Helms AW , Kim EJ , Savage TK , Lagace DC , Mandyam CD , Eisch AJ , Miyoshi G , Johnson JE. 2007 Ascl1 defines sequentially generated lineage‐restricted neuronal and oligodendrocyte precursor cells in the spinal cord. Development 134:285–293. 17166924 10.1242/dev.02727

[glia22850-bib-0009] Beckervordersandforth R , Tripathi P , Ninkovic J , Bayam E , Lepier A , Stempfhuber B , Kirchhoff F , Hirrlinger J , Haslinger A , Lie DC , Beckers J , Yoder B , Irmler M , Götz M. 2010 In vivo fate mapping and expression analysis reveals molecular hallmarks of prospectively isolated adult neural stem cells. Cell Stem Cell 7:744–758. 21112568 10.1016/j.stem.2010.11.017

[glia22850-bib-0010] Benner EJ , Luciano D , Jo R , Abdi K , Paez‐Gonzalez P , Sheng H , Warner DS , Liu C , Eroglu C , Kuo CT. 2013 Protective astrogenesis from the SVZ niche after injury is controlled by Notch modulator Thbs4. Nature 497:369–373. 23615612 10.1038/nature12069 PMC3667629

[glia22850-bib-0011] Bi B , Salmaso N , Komitova M , Simonini MV , Silbereis J , Cheng E , Kim J , Luft S , Ment LR , Horvath TL , Schwartz ML , Vaccarino FM. 2011 Cortical glial fibrillary acidic protein‐positive cells generate neurons after perinatal hypoxic injury. J Neurosci 31:9205–9021. 21697371 10.1523/JNEUROSCI.0518-11.2011 PMC3142780

[glia22850-bib-0012] Bonaguidi MA , Wheeler MA , Shapiro JS , Stadel RP , Sun GJ , Ming GL , Song H. 2011 In vivo clonal analysis reveals self‐renewing and multipotent adult neural stem cell characteristics. Cell 145:1142–1155. 21664664 10.1016/j.cell.2011.05.024 PMC3124562

[glia22850-bib-0013] Borrell V , Götz M. 2014 Role of radial glial cells in cerebral cortex folding. Curr Opin Neurobiol 27:39–46. 24632307 10.1016/j.conb.2014.02.007

[glia22850-bib-0014] Brill MS , Ninkovic J , Winpenny E , Hodge RD , Ozen I , Yang R , Lepier A , Gascón S , Erdelyi F , Szabo G , Parras C , Guillemot F , Frotscher M , Berninger B , Hevner RF , Raineteau O , Götz M. 2009 Adult generation of glutamatergic olfactory bulb interneurons. Nat Neurosci 12:1524–1533. 19881504 10.1038/nn.2416 PMC2787799

[glia22850-bib-0015] Brilli E , Reitano E , Conti L , Conforti P , Gulino R , Consalez GG , Cesana E , Smith A , Rossi F , Cattaneo E. 2013 Neural stem cells engrafted in the adult brain fuse with endogenous neurons. Stem Cells Dev 22:538–547. 23009360 10.1089/scd.2012.0530

[glia22850-bib-0016] Bruni JE. 1998 Ependymal development, proliferation, and functions: A review. Microsc Res Tech 41:2–13. 9550133 10.1002/(SICI)1097-0029(19980401)41:1<2::AID-JEMT2>3.0.CO;2-Z

[glia22850-bib-0017] Buffo A , Rite I , Tripathi P , Lepier A , Colak D , Horn AP , Mori T , Götz M. 2008 Origin and progeny of reactive gliosis: A source of multipotent cells in the injured brain. Proc Natl Acad Sci USA 105:3581–3586 18299565 10.1073/pnas.0709002105 PMC2265175

[glia22850-bib-0018] Busch K , Klapproth K , Barile M , Flossdorf M , Holland‐Letz T , Schlenner SM , Reth M , Höfer T , Rodewald HR. 2015 Fundamental properties of unperturbed haematopoiesis from stem cells in vivo. Nature 518:542–546. 25686605 10.1038/nature14242

[glia22850-bib-0019] Cahoy JD , Emery B , Kaushal A , Foo LC , Zamanian JL , Christopherson KS , Xing Y , Lubischer JL , Krieg PA , Krupenko SA , Thompson WJ , Barres BA. 2008 A transcriptome database for astrocytes, neurons, and oligodendrocytes: A new resource for understanding brain development andfunction. J Neurosci 28:264–278. 18171944 10.1523/JNEUROSCI.4178-07.2008 PMC6671143

[glia22850-bib-0020] Calzolari F , Michel J , Baumgart EV , Theis F , Götz M , Ninkovic J. 2015 Fast clonal expansion and limited neural stem cell self‐renewal in the adult subependymal zone. Nat Neurosci 18:490–492. 25730673 10.1038/nn.3963

[glia22850-bib-0021] Caputi A , Melzer S , Michael M , Monyer H. 2013 The long and short of GABAergic neurons. Curr Opin Neurobiol 23:179–186. 23394773 10.1016/j.conb.2013.01.021

[glia22850-bib-0022] Carlén M , Meletis K , Göritz C , Darsalia V , Evergren E , Tanigaki K , Amendola M , Barnabé‐Heider F , Yeung MS , Naldini L , Honjo T , Kokaia Z , Shupliakov O , Cassidy RM , Lindvall O , Frisén J. 2009 Forebrain ependymal cells are Notch‐dependent and generate neuroblasts and astrocytes after stroke. Nat Neurosci 12:259–267. 19234458 10.1038/nn.2268

[glia22850-bib-0023] Chen X , Lepier A , Berninger B , Tolkovsky AM , Herbert J. 2012 Cultured subventricular zone progenitor cells transduced with neurogenin‐2 become mature glutamatergic neurons and integrate into the dentate gyrus. PLoS One 7:e31547. 22348101 10.1371/journal.pone.0031547 PMC3279376

[glia22850-bib-0024] Clapes T , Robin C. 2012 Embryonic development of hematopoietic stem cells: Implications for clinical use. Regen Med 7:349–368. 22594328 10.2217/rme.11.120

[glia22850-bib-0025] Codega P , Silva‐Vargas V , Paul A , Maldonado‐Soto AR , Deleo AM , Pastrana E , Doetsch F. 2014 Prospective identification and purification of quiescent adult neural stem cells from their in vivo niche. Neuron 82:545–559. 24811379 10.1016/j.neuron.2014.02.039 PMC4360885

[glia22850-bib-0026] Costa MR , Ortega F , Brill MS , Beckervordersandforth R , Petrone C , Schroeder T , Götz M , Berninger B. 2011 Continuous live imaging of adult neural stem cell division and lineage progression in vitro. Development 138:1057–1068. 21343361 10.1242/dev.061663

[glia22850-bib-0027] Curtis MA , Eriksson PS , Faull RL. 2007 Progenitor cells and adult neurogenesis in neurodegenerative diseases and injuries of the basal ganglia. Clin Exp Pharmacol Physiol 34:528–532. 17439428 10.1111/j.1440-1681.2007.04609.x

[glia22850-bib-0028] De Juan Romero C , Borrell V. 2015. Co‐evolution of radial glial cells and the cerebral cortex. Glia (this issue). 10.1002/glia.22827 PMC5008138 25808466

[glia22850-bib-0029] Desai AR , McConnell SK. 2000 Progressive restriction in fate potential by neural progenitors during cerebral cortical development. Development 127:2863–2872. 10851131 10.1242/dev.127.13.2863

[glia22850-bib-0030] Dimou L , Gallo V. 2015. NG2‐glia and their functions in the central nervous system. Glia (this issue). 10.1002/glia.22859 PMC4470768 26010717

[glia22850-bib-0031] Dimou L , Götz M. 2014 Glial cells as progenitors and stem cells: New roles in the healthy and diseased brain. Physiol Rev 94:709–737. 24987003 10.1152/physrev.00036.2013

[glia22850-bib-0032] Dimou L , Simon C , Kirchhoff F , Takebayashi H , Götz M. 2008 Progeny of Olig2‐expressing progenitors in the gray and white matter of the adult mouse cerebral cortex. J Neurosci 28:10434–10442. 18842903 10.1523/JNEUROSCI.2831-08.2008 PMC6671038

[glia22850-bib-0033] Encinas JM , Michurina TV , Peunova N , Park JH , Tordo J , Peterson DA , Fishell G , Koulakov A , Enikolopov G. 2011 Division‐coupled astrocytic differentiation and age‐related depletion of neural stem cells in the adult hippocampus. Cell Stem Cell 8:566–579. 21549330 10.1016/j.stem.2011.03.010 PMC3286186

[glia22850-bib-0034] Ernst A , Alkass K , Bernard S , Salehpour M , Perl S , Tisdale J , Possnert G , Druid HJ. 2014 Neurogenesis in the striatum of the adult human brain. Cell 156:1072–1083. 24561062 10.1016/j.cell.2014.01.044

[glia22850-bib-0035] Etxeberria A , Mangin JM , Aguirre A , Gallo V. 2010 Adult‐born SVZ progenitors receive transient synapses during remyelination in corpus callosum. Nat Neurosci 13:287–289. 20173746 10.1038/nn.2500 PMC4681435

[glia22850-bib-0036] Fischer J , Beckervordersandforth R , Tripathi P , Steiner‐Mezzadri A , Ninkovic J , Götz M. 2011 Prospective isolation of adult neural stem cells from the mouse subependymal zone. Nat Protoc 6:1981–1989. 22094733 10.1038/nprot.2011.412

[glia22850-bib-0037] Frantz GD , McConnell SK. 1996 Restriction of late cerebral cortical progenitors to an upper‐layer fate. Neuron 17:55–61. 8755478 10.1016/s0896-6273(00)80280-9

[glia22850-bib-0165] Furutachi S , Miya H , Watanabe T , Kawai H , Yamasaki N , Harada Y , Imayoshi I , Nelson M , Nakayama KI , Hirabayashi Y , Gotoh Y . 2015 Slowly dividing neural progenitors are an embryonic origin of adult neural stem cells. Nat Neurosci doi: 10.1038/nn.3989. 10.1038/nn.3989 25821910

[glia22850-bib-0038] Gabay L , Lowell S , Rubin LL , Anderson DJ. 2003 Deregulation of dorsoventral patterning by FGF confers trilineage differentiation capacity on CNS stem cells in vitro. Neuron 40:485–499. 14642274 10.1016/s0896-6273(03)00637-8

[glia22850-bib-0039] Gage FH , Kempermann G , Palmer TD , Peterson DA , Ray J. 1998 Multipotent progenitor cells in the adult dentate gyrus. J Neurobiol 36:249–266. 9712308 10.1002/(sici)1097-4695(199808)36:2<249::aid-neu11>3.0.co;2-9

[glia22850-bib-0040] Gage FH , Ray J , Fisher LJ. 1995 Isolation, characterization, and use of stem cells from the CNS. Annu Rev Neurosci 18:159–192. 7605059 10.1146/annurev.ne.18.030195.001111

[glia22850-bib-0041] Ganat YM , Silbereis J , Cave C , Ngu H , Anderson GM , Ohkubo Y , Ment LR , Vaccarino FM. 2006 Early postnatal astroglial cells produce multilineage precursors and neural stem cells in vivo. J Neurosci 26:8609–8621. 16914687 10.1523/JNEUROSCI.2532-06.2006 PMC6674357

[glia22850-bib-0042] Gao P , Postiglione MP , Krieger TG , Hernandez L , Wang C , Han Z , Streicher C , Papusheva E , Insolera R , Chugh K , Kodish O , Huang K , Simons BD , Luo L , Hippenmeyer S , Shi SH. 2014 Deterministic progenitor behavior and unitary production of neurons in the neocortex. Cell 159:775–788. 25417155 10.1016/j.cell.2014.10.027 PMC4225456

[glia22850-bib-0043] García‐Marqués J , López‐Mascaraque L. 2013 Clonal identity determines astrocyte cortical heterogeneity. Cereb Cortex 23:1463–1472. 22617854 10.1093/cercor/bhs134

[glia22850-bib-0044] Ge WP , Miyawaki A , Gage FH , Jan YN , Jan LY. 2012 Local generation of glia is a major astrocyte source in postnatal cortex. Nature 484:376–380. 22456708 10.1038/nature10959 PMC3777276

[glia22850-bib-0046] Götz M. 2013 Radial glial cells In: K Helmut, R Bruce R, editors. Neuroglia, 3rd ed New York: Oxford University Press pp 50–61.

[glia22850-bib-0047] Götz M , Huttner WB. 2005 The cell biology of neurogenesis. Nat Rev Mol Cell Biol 6:777–788. 16314867 10.1038/nrm1739

[glia22850-bib-0048] Götz M , Sirko S. 2013 Potential of glial cells In: Sell Stewart, editor. Stem cells handbook, 2nd ed New York: Springer pp 347–361.

[glia22850-bib-0049] Grande A , Sumiyoshi K , López‐Juárez A , Howard J , Sakthivel B , Aronow B , Campbell K , Nakafuku M. 2013 Environmental impact on direct neuronal reprogramming in vivo in the adult brain. Nat Commun 4:2373. 23974433 10.1038/ncomms3373 PMC3786770

[glia22850-bib-0050] Grégoire CA , Goldenstein BL , Floriddia EM , Barnabé‐Heider F , Fernandes KJL. 2015. Endogenous neural stem cell responses to stroke and spinal cord injury. Glia (this issue). 10.1002/glia.22851 25921491

[glia22850-bib-0051] Greig LC , Woodworth MB , Galazo MJ , Padmanabhan H , Macklis JD. 2013 Molecular logic of neocortical projection neuron specification, development and diversity. Nat Rev Neurosci 14:755–769. 24105342 10.1038/nrn3586 PMC3876965

[glia22850-bib-0052] Grove EA , Williams BP , Li DQ , Hajihosseini M , Friedrich A , Price J. 1993 Multiple restricted lineages in the embryonic rat cerebral cortex. Development 117:553–561. 8330526 10.1242/dev.117.2.553

[glia22850-bib-0053] Guérout N , Li X , Barnabé‐Heider F. 2014 Cell fate control in the developing central nervous system. Exp Cell Res 321:77–83. 24140262 10.1016/j.yexcr.2013.10.003

[glia22850-bib-0054] Haan N , Goodman T , Najdi‐Samiei A , Stratford CM , Rice R , El Agha E , Bellusci S , Hajihosseini MK. 2013 Fgf10‐expressing tanycytes add new neurons to the appetite/energy‐balance regulating centers of the postnatal and adult hypothalamus. J Neurosci 33:6170–6180. 23554498 10.1523/JNEUROSCI.2437-12.2013 PMC3736310

[glia22850-bib-0055] Hack MA , Sugimori M , Lundberg C , Nakafuku M , Götz M. 2004 Regionalization and fate specification in neurospheres: The role of Olig2 and Pax6. Mol Cell Neurosci 25:664–678. 15080895 10.1016/j.mcn.2003.12.012

[glia22850-bib-0056] Hartfuss E , Galli R , Heins N , Götz M. 2001 Characterization of CNS precursor subtypes and radial glia. Dev Biol 229:15–30. 11133151 10.1006/dbio.2000.9962

[glia22850-bib-0057] Hochstim C , Deneen B , Lukaszewicz A , Zhou Q , Anderson DJ. 2008 Identification of positionally distinct astrocyte subtypes whose identities are specified by a homeodomain code. Cell 133:510–522. 18455991 10.1016/j.cell.2008.02.046 PMC2394859

[glia22850-bib-0058] Hsu YC , Li L , Fuchs E. 2014 Emerging interactions between skin stem cells and their niches. Nat Med 20:847–856. 25100530 10.1038/nm.3643 PMC4358898

[glia22850-bib-0059] Ihrie RA , Shah JK , Harwell CC , Levine JH , Guinto CD , Lezameta M , Kriegstein AR , Alvarez‐Buylla A. 2011 Persistent sonic hedgehog signaling in adult brain determines neural stem cell positional identity. Neuron 71:250–262. 21791285 10.1016/j.neuron.2011.05.018 PMC3346180

[glia22850-bib-0060] Imayoshi I , Kageyama R. 2014 Oscillatory control of bHLH factors in neural progenitors. Trends Neurosci 37:531–538. 25149265 10.1016/j.tins.2014.07.006

[glia22850-bib-0061] Inta D , Alfonso J , von Engelhardt J , Kreuzberg MM , Meyer AH , van Hooft JA , Monyer H. 2008 Neurogenesis and widespread forebrain migration of distinct GABAergic neurons from the postnatal subventricular zone. Proc Natl Acad Sci USA 105:20994–20999. 19095802 10.1073/pnas.0807059105 PMC2605417

[glia22850-bib-0062] Jacquet BV , Salinas‐Mondragon R , Liang H , Therit B , Buie JD , Dykstra M , Campbell K , Ostrowski LE , Brody SL , Ghashghaei HT. 2009 FoxJ1‐dependent gene expression is required for differentiation of radial glia into ependymal cells and a subset of astrocytes in the postnatal brain. Development 136:4021–4031. 19906869 10.1242/dev.041129 PMC3118431

[glia22850-bib-0063] Jiménez AJ , Domínguez‐Pinos MD , Guerra MM , Fernández‐Llebrez P , Pérez‐Fígares JM. 2014 Structure and function of the ependymal barrier and diseases associated with ependyma disruption. Tissue Barriers 2:e28426. 25045600 10.4161/tisb.28426 PMC4091052

[glia22850-bib-0064] Kang W , Balordi F , Su N , Chen L , Fishell G , Hébert JM. 2014 Astrocyte activation is suppressed in both normal and injured brain by FGF signaling. Proc Natl Acad Sci USA 111:E2987–E2995. 25002516 10.1073/pnas.1320401111 PMC4115557

[glia22850-bib-0065] Kazanis I , Lathia J , Moss L , ffrench‐Constant C . 2008 The neural stem cell microenvironment In: The Stem CellStemBook [Internet]. Cambridge (MA): Harvard Stem Cell Institute Doi: 10.3824/stembook.1.15.1 http://www.stembook.org/node/490. 20614585

[glia22850-bib-0066] Kondo T , Raff M. 2000 Oligodendrocyte precursor cells reprogrammed to become multipotential CNS stem cells. Science 289:1754–1757. 10976069 10.1126/science.289.5485.1754

[glia22850-bib-0067] Kriegstein A , Alvarez‐Buylla A. 2009 The glial nature of embryonic and adult neural stem cells. Annu Rev Neurosci 32:149–184. 19555289 10.1146/annurev.neuro.051508.135600 PMC3086722

[glia22850-bib-0068] Ledford H. 2008 Disputed definitions. Nature 455:1023–1028. 18948925 10.1038/4551023a

[glia22850-bib-0069] Le Magueresse C , Alfonso J , Khodosevich K , Arroyo Martín AA , Bark C , Monyer H. 2011 “Small axonless neurons”: Postnatally generated neocortical interneurons with delayed functional maturation. J Neurosci 31:16731–16747. 22090500 10.1523/JNEUROSCI.4273-11.2011 PMC6633314

[glia22850-bib-0070] Leone DP , Srinivasan K , Chen B , Alcamo E , McConnell SK. 2008 The determination of projection neuron identity in the developing cerebral cortex. Curr Opin Neurobiol 18:28–35. 18508260 10.1016/j.conb.2008.05.006 PMC2483251

[glia22850-bib-0071] Levison SW , Goldman JE. 1993 Both oligodendrocytes and astrocytes develop from progenitors in the subventricular zone of postnatal rat forebrain. Neuron 10:201–212. 8439409 10.1016/0896-6273(93)90311-e

[glia22850-bib-0072] Levison SW , Goldman JE. 1997 Multipotential and lineage restricted precursors coexist in the mammalian perinatal subventricular zone. J Neurosci Res 48:83–94. 9130137

[glia22850-bib-0073] Li H , de Faria JP , Andrew P , Nitarska J , Richardson WD. 2011 Phosphorylation regulates OLIG2 cofactor choice and the motor neuron‐oligodendrocyte fate switch. Neuron 69:918–929. 21382552 10.1016/j.neuron.2011.01.030 PMC3093612

[glia22850-bib-0074] Lin G , Goldman JE. 2009 An FGF‐responsive astrocyte precursor isolated from the neonatal forebrain. Glia 57:592–603. 19031440 10.1002/glia.20788 PMC2657186

[glia22850-bib-0075] Lledo PM , Merkle FT , Alvarez‐Buylla A. 2008 Origin and function of olfactory bulb interneuron diversity. Trends Neurosci 31:392–400. 18603310 10.1016/j.tins.2008.05.006 PMC4059175

[glia22850-bib-0076] Lodato S , Shetty AS , Arlotta P. 2015 Cerebral cortex assembly: Generating and reprogramming projection neuron diversity. Trends Neurosci 38:117–125. 25529141 10.1016/j.tins.2014.11.003 PMC4334136

[glia22850-bib-0077] Luskin MB. 1993 Restricted proliferation and migration of postnatally generated neurons derived from the forebrain subventricular zone. Neuron 11:173–189. 8338665 10.1016/0896-6273(93)90281-u

[glia22850-bib-0078] Luzzati F , De Marchis S , Fasolo A , Peretto P. 2006 Neurogenesis in the caudate nucleus of the adult rabbit. J Neurosci 26:609–621. 16407559 10.1523/JNEUROSCI.4371-05.2006 PMC6674396

[glia22850-bib-0079] Magnusson JP , Göritz C , Tatarishvili J , Dias DO , Smith EM , Lindvall O , Kokaia Z , Frisén J. 2014 A latent neurogenic program in astrocytes regulated by Notch signaling in the mouse. Science 346:237–241. 25301628 10.1126/science.346.6206.237

[glia22850-bib-0080] Malatesta P , Hack MA , Hartfuss E , Kettenmann H , Klinkert W , Kirchhoff F , Götz M. 2003 Neuronal or glial progeny: Regional differences in radial glia fate. Neuron 37:751–764. 12628166 10.1016/s0896-6273(03)00116-8

[glia22850-bib-0081] Malatesta P , Hartfuss E , Götz M. 2000 Isolation of radial glial cells by fluorescent‐activated cell sorting reveals a neuronal lineage. Development 127:5253–5263. 11076748 10.1242/dev.127.24.5253

[glia22850-bib-0082] Marshall CA , Novitch BG , Goldman JE. 2005 Olig2 directs astrocyte and oligodendrocyte formation in postnatal subventricular zone cells. J Neurosci 25:7289–7298. 16093378 10.1523/JNEUROSCI.1924-05.2005 PMC6725308

[glia22850-bib-0083] Marshall CA , Suzuki SO , Goldman JE. 2003 Gliogenic and neurogenic progenitors of the subventricular zone: Who are they, where did they come from, and where are they going? Glia 43:52–61. 12761867 10.1002/glia.10213

[glia22850-bib-0084] Martín‐López E , García‐Marques J , Núñez‐Llaves R , López‐Mascaraque L. 2013 Clonal astrocytic response to cortical injury. PLoS One 8:e74039. 24040158 10.1371/journal.pone.0074039 PMC3769363

[glia22850-bib-0085] Martynoga B , Mateo JL , Zhou B , Andersen J , Achimastou A , Urbán N , van den Berg D , Georgopoulou D , Hadjur S , Wittbrodt J , Ettwiller L , Piper M , Gronostajski RM , Guillemot F. 2013 Epigenomic enhancer annotation reveals a key role for NFIX in neural stem cell quiescence. Genes Dev 27:1769–1786. 23964093 10.1101/gad.216804.113 PMC3759694

[glia22850-bib-0086] McDermott KW , Barry DS , McMahon SS. 2005 Role of radial glia in cytogenesis, patterning and boundary formation in the developing spinal cord. J Anat 207:241–250. 16185248 10.1111/j.1469-7580.2005.00462.x PMC1571535

[glia22850-bib-0087] Meletis K , Barnabé‐Heider F , Carlén M , Evergren E , Tomilin N , Shupliakov O , Frisén J. 2008 Spinal cord injury reveals multilineage differentiation of ependymal cells. PLoS Biol 6:e182. 18651793 10.1371/journal.pbio.0060182 PMC2475541

[glia22850-bib-0088] Merkle FT , Fuentealba LC , Sanders TA , Magno L , Kessaris N , Alvarez‐Buylla A. 2014 Adult neural stem cells in distinct microdomains generate previously unknown interneuron types. Nat Neurosci 17:207–214. 24362763 10.1038/nn.3610 PMC4100623

[glia22850-bib-0089] Merkle FT , Mirzadeh Z , Alvarez‐Buylla A. 2007 Mosaic organization of neural stem cells in the adult brain. Science 317:381–384. 17615304 10.1126/science.1144914

[glia22850-bib-0090] Merkle FT , Tramontin AD , Garcia‐Verdugo JM , Alvarez‐Buylla A. 2004 Radial glia give rise to adult neural stem cells in the subventricular zone. Proc Natl Acad Sci USA 101:17528–17532. 15574494 10.1073/pnas.0407893101 PMC536036

[glia22850-bib-0091] Mich JK , Signer RA , Nakada D , Pineda A , Burgess RJ , Vue TY , Johnson JE , Morrison SJ. 2014 Prospective identification of functionally distinct stem cells and neurosphere‐initiating cells in adult mouse forebrain. Elife 3:e02669. 24843006 10.7554/eLife.02669 PMC4038845

[glia22850-bib-0092] Molofsky AV , Deneen B. 2015. Astrocyte development: A guide for the perplexed. Glia (this issue). 10.1002/glia.22836 25963996

[glia22850-bib-0093] Molofsky AV , Kelley KW , Tsai HH , Redmond SA , Chang SM , Madireddy L , Chan JR , Baranzini SE , Ullian EM , Rowitch DH. 2014 Astrocyte‐encoded positional cues maintain sensorimotor circuit integrity. Nature 509:189‐194. 24776795 10.1038/nature13161 PMC4057936

[glia22850-bib-0095] Mukouyama YS , Deneen B , Lukaszewicz A , Novitch BG , Wichterle H , Jessell TM , Anderson DJ. 2006 Olig2+ neuroepithelial motoneuron progenitors are not multipotent stem cells in vivo. Proc Natl Acad Sci USA 103:1551–1556. 16432183 10.1073/pnas.0510658103 PMC1345718

[glia22850-bib-0096] Nagy A , Gócza E , Diaz EM , Prideaux VR , Iványi E , Markkula M , Rossant J. 1990 Embryonic stem cells alone are able to support fetal development in the mouse. Development 110:815–821. 2088722 10.1242/dev.110.3.815

[glia22850-bib-0097] Nakatomi H , Kuriu T , Okabe S , Yamamoto S , Hatano O , Kawahara N , Tamura A , Kirino T , Nakafuku M. 2002 Regeneration of hippocampal pyramidal neurons after ischemic brain injury by recruitment of endogenous neural progenitors. Cell 110:429–441. 12202033 10.1016/s0092-8674(02)00862-0

[glia22850-bib-0098] Neumeister B , Grabosch A , Basak O , Kemler R , Taylor V. 2009 Neural progenitors of the postnatal and adult mouse forebrain retain the ability to self‐replicate, form neurospheres, and undergo multipotent differentiation in vivo. Stem Cells 27:714–723. 19096037 10.1634/stemcells.2008-0985

[glia22850-bib-0099] Ninkovic J , Götz M. 2014 A time and place for understanding neural stem cell specification. Dev Cell 30:114–115. 25073151 10.1016/j.devcel.2014.06.023

[glia22850-bib-0100] Ninkovic J , Götz M. 2015 How to make neurons‐thoughts on the molecular logic of neurogenesis in the central nervous system. Cell Tissue Res 359:5–16. 25416507 10.1007/s00441-014-2048-9

[glia22850-bib-0101] Ninkovic J , Mori T , Götz M. 2007 Distinct modes of neuron addition in adult mouse neurogenesis. J Neurosci 27:10906–10911. 17913924 10.1523/JNEUROSCI.2572-07.2007 PMC6672836

[glia22850-bib-0102] Ninkovic J , Steiner‐Mezzadri A , Jawerka M , Akinci U , Masserdotti G , Petricca S , Fischer J , von Holst A , Beckers J , Lie CD , Petrik D , Miller E , Tang J , Wu J , Lefebvre V , Demmers J , Eisch A , Metzger D , Crabtree G , Irmler M , Poot R , Götz M. 2013 The BAF complex interacts with Pax6 in adult neural progenitors to establish a neurogenic cross‐regulatory transcriptional network. Cell Stem Cell 13:403–418. 23933087 10.1016/j.stem.2013.07.002 PMC4098720

[glia22850-bib-0103] Nishiyama A , Komitova M , Suzuki R , Zhu X. 2009 Polydendrocytes (NG2 cells): Multifunctional cells with lineage plasticity. Nature Rev Neurosci 10:9–22. 19096367 10.1038/nrn2495

[glia22850-bib-0104] Noctor SC , Martínez‐Cerdeño V , Ivic L , Kriegstein AR. 2004 Cortical neurons arise in symmetric and asymmetric division zones and migrate through specific phases. Nat Neurosci 7:136–144. 14703572 10.1038/nn1172

[glia22850-bib-0105] Nolte C , Matyash M , Pivneva T , Schipke CG , Ohlemeyer C , Hanisch UK , Kirchhoff F , Kettenmann H. 2001 GFAP promoter‐controlled EGFP‐expressing transgenic mice: A tool to visualize astrocytes and astrogliosis in living brain tissue. Glia 33:72–86. 11169793

[glia22850-bib-0106] Norton WT , Aquino DA , Hozumi I , Chiu FC , Brosnan CF. 1992 Quantitative aspects of reactive gliosis: A review. Neurochem Res 17:877–885. 1407275 10.1007/BF00993263

[glia22850-bib-0107] Ohori Y , Yamamoto S , Nagao M , Sugimori M , Yamamoto N , Nakamura K , Nakafuku M. 2006 Growth factor treatment and genetic manipulation stimulate neurogenesis and oligodendrogenesis by endogenous neural progenitors in the injured adult spinal cord. J Neurosci 26:11948–11960. 17108169 10.1523/JNEUROSCI.3127-06.2006 PMC6674878

[glia22850-bib-0108] Orre M , Kamphuis W , Osborn LM , Melief J , Kooijman L , Huitinga I , Klooster J , Bossers K , Hol EM. 2014 Acute isolation and transcriptome characterization of cortical astrocytes and microglia from young and aged mice. Neurobiol Aging 35:1–14. 23954174 10.1016/j.neurobiolaging.2013.07.008

[glia22850-bib-0109] Ortega F , Gascón S , Masserdotti G , Deshpande A , Simon C , Fischer J , Dimou L , Chichung Lie D , Schroeder T , Berninger B. 2013 Oligodendrogliogenic and neurogenic adult subependymal zone neural stem cells constitute distinct lineages and exhibit differential responsiveness to Wnt ignaling. Nat Cell Biol 15:602–613. 23644466 10.1038/ncb2736

[glia22850-bib-0110] Paez‐Gonzalez P , Abdi K , Luciano D , Liu Y , Soriano‐Navarro M , Rawlins E , Bennett V , Garcia‐Verdugo JM , Kuo CT. 2011 Ank3‐dependent SVZ niche assembly is required for the continued production of new neurons. Neuron 71:61–75. 21745638 10.1016/j.neuron.2011.05.029 PMC3134799

[glia22850-bib-0111] Palmer TD , Markakis EA , Willhoite AR , Safar F , Gage FH. 1999 Fibroblast growth factor‐2 activates a latent neurogenic program in neural stem cells from diverse regions of the adult CNS. J Neurosci 19:8487–8497. 10493749 10.1523/JNEUROSCI.19-19-08487.1999 PMC6783019

[glia22850-bib-0112] Pekny M , Johansson CB , Eliasson C , Stakeberg J , Wallén A , Perlmann T , Lendahl U , Betsholtz C , Berthold CH , Frisén J. 1999 Abnormal reaction to central nervous system injury in mice lacking glial fibrillary acidic protein and vimentin. J Cell Biol 145:503–514. 10225952 10.1083/jcb.145.3.503 PMC2185074

[glia22850-bib-0113] Pekny M , Pekna M. 2014 Astrocyte reactivity and reactive astrogliosis: Costs and benefits. Physiol Rev 94:1077–1098. 25287860 10.1152/physrev.00041.2013

[glia22850-bib-0114] Pilz GA , Shitamukai A , Reillo I , Pacary E , Schwausch J , Stahl R , Ninkovic J , Snippert HJ , Clevers H , Godinho L , Guillemot F , Borrell V , Matsuzaki F , Götz M. 2013 Amplification of progenitors in the mammalian telencephalon includes a new radial glial cell type. Nat Commun 4:2125. 23839311 10.1038/ncomms3125 PMC3717501

[glia22850-bib-0115] Pinto L , Drechsel D , Schmid MT , Ninkovic J , Irmler M , Brill MS , Restani L , Gianfranceschi L , Cerri C , Weber SN , Tarabykin V , Baer K , Guillemot F , Beckers J , Zecevic N , Dehay C , Caleo M , Schorle H , Götz M. 2009 AP2gamma regulates basal progenitor fate in a region‐ and layer‐specific manner in the developing cortex. Nat Neurosci 12:1229–1237. 19749747 10.1038/nn.2399

[glia22850-bib-0116] Pinto L , Mader MT , Irmler M , Gentilini M , Santoni F , Drechsel D , Blum R , Stahl R , Bulfone A , Malatesta P , Beckers J , Götz M. 2008 Prospective isolation of functionally distinct radial glial subtypes–lineage and transcriptome analysis. Mol Cell Neurosci 38:15–42. 18372191 10.1016/j.mcn.2008.01.012

[glia22850-bib-0117] Pollard SM , Conti L , Sun Y , Goffredo D , Smith A. 2006 Adherent neural stem (NS) cells from fetal and adult forebrain. Cereb Cortex 16 Suppl 1:i112–i120. 16766697 10.1093/cercor/bhj167

[glia22850-bib-0118] Pollard SM , Wallbank R , Tomlinson S , Grotewold L , Smith A. 2008 Fibroblast growth factor induces a neural stem cell phenotype in foetal forebrain progenitors and during embryonic stem cell differentiation. Mol Cell Neurosci 38:393–403. 18504136 10.1016/j.mcn.2008.03.012

[glia22850-bib-0119] Psachoulia K , Jamen F , Young KM , Richardson WD. 2009 Cell cycle dynamics of NG2 cells in the postnatal and ageing brain. Neuron Glia Biol 5:57–67 20346197 10.1017/S1740925X09990354 PMC6329448

[glia22850-bib-0120] Qian X , Shen Q , Goderie SK , He W , Capela A , Davis AA , Temple S. 2000 Timing of CNS cell generation: A programmed sequence of neuron and glial cell production from isolated murine cortical stem cells. Neuron 28:69–80. 11086984 10.1016/s0896-6273(00)00086-6

[glia22850-bib-0121] Reynolds BA , Weiss S. 1992 Generation of neurons and astrocytes from isolated cells of the adult mammalian central nervous system. Science 255:1707–1710. 1553558 10.1126/science.1553558

[glia22850-bib-0122] Reynolds R , Hardy R. 1997 Oligodendroglial progenitors labeled with the O4 antibody persist in the adult rat cerebral cortex in vivo. J Neurosci Res 47:455–470. 9067855 10.1002/(sici)1097-4547(19970301)47:5<455::aid-jnr1>3.0.co;2-g

[glia22850-bib-0123] Richards LJ , Kilpatrick TJ , Bartlett PF. 1992 De novo generation of neuronal cells from the adult mouse brain. Proc Natl Acad Sci USA 89:8591–8595. 1528866 10.1073/pnas.89.18.8591 PMC49966

[glia22850-bib-0124] Riquelme PA , Drapeau E , Doetsch F. 2008 Brain micro‐ecologies: Neural stem cell niches in the adult mammalian brain. Philos Trans R Soc Lond B Biol Sci 363:123–137. 17322003 10.1098/rstb.2006.2016 PMC2605490

[glia22850-bib-0125] Robel S , Berninger B , Götz M. 2011 The stem cell potential of glia: Lessons from reactive gliosis. Nat Rev Neurosci 12:88–104. 21248788 10.1038/nrn2978

[glia22850-bib-0126] Robins SC , Stewart I , McNay DE , Taylor V , Giachino C , Goetz M , Ninkovic J , Briancon N , Maratos‐Flier E , Flier JS , Kokoeva MV , Placzek M. 2013 Α‐Tanycytes of the adult hypothalamic third ventricle include distinct populations of FGF‐responsive neural progenitors. Nat Commun 4:2049. 23804023 10.1038/ncomms3049

[glia22850-bib-0127] Rousso DL , Pearson CA , Gaber ZB , Miquelajauregui A , Li S , Portera‐Cailliau C , Morrisey EE , Novitch BG. 2012 Foxp‐mediated suppression of N‐cadherin regulates neuroepithelial character and progenitor maintenance in the CNS. Neuron 74:314–330. 22542185 10.1016/j.neuron.2012.02.024 PMC3444171

[glia22850-bib-0128] Rowitch DH , Kriegstein AR. 2010 Developmental genetics of vertebrate glial‐cell specification. Nature 468:214–222. 21068830 10.1038/nature09611

[glia22850-bib-0129] Sabelström H , Stenudd M , Réu P , Dias DO , Elfineh M , Zdunek S , Damberg P , Göritz C , Frisén J. 2013 Resident neural stem cells restrict tissue damage and neuronal loss after spinal cord injury in mice. Science 342:637–640. 24179227 10.1126/science.1242576

[glia22850-bib-0130] Sahara S , O'Leary DD. 2009 Fgf10 regulates transition period of cortical stem cell differentiation to radial glia controlling generation of neurons and basal progenitors. Neuron 63:48–62. 19607792 10.1016/j.neuron.2009.06.006 PMC2746711

[glia22850-bib-0131] Seidenfaden R , Desoeuvre A , Bosio A , Virard I , Cremer H. 2006 Glial conversion of SVZ‐derived committed neuronal precursors after ectopic grafting into the adult brain. Mol Cell Neurosci 32:187–198. 16730456 10.1016/j.mcn.2006.04.003

[glia22850-bib-0132] Shear DA , Tate MC , Archer DR , Hoffman SW , Hulce VD , Laplaca MC , Stein DG. 2004 Neural progenitor cell transplants promote long‐term functional recovery after traumatic brain injury. Brain Res 1026:11–22. 15476693 10.1016/j.brainres.2004.07.087

[glia22850-bib-0133] Shihabuddin LS , Horner PJ , Ray J , Gage FH. 2000 Adult spinal cord stem cells generate neurons after transplantation in the adult dentate gyrus. J Neurosci 20:8727–8735. 11102479 10.1523/JNEUROSCI.20-23-08727.2000 PMC6773057

[glia22850-bib-0134] Shimada IS , LeComte MD , Granger JC , Quinlan NJ , Spees JL. 2012 Self‐renewal and differentiation of reactive astrocyte‐derived neural stem/progenitor cells isolated from the cortical peri‐infarct area after stroke. J Neurosci 32:7926–7940. 22674268 10.1523/JNEUROSCI.4303-11.2012 PMC3398807

[glia22850-bib-0135] Shimojo H , Ohtsuka T , Kageyama R. 2011 Dynamic expression of notch signaling genes in neural stem/progenitor cells. Front Neurosci 5:78. 21716644 10.3389/fnins.2011.00078 PMC3116140

[glia22850-bib-0136] Simon C , Götz M , Dimou L. 2011 Progenitors in the adult cerebral cortex: Cell cycle properties and regulation by physiological stimuli and injury. Glia 59:869–881. 21446038 10.1002/glia.21156

[glia22850-bib-0137] Sirko S , Behrendt G , Johansson PA , Tripathi P , Costa M , Bek S , Heinrich C , Tiedt S , Colak D , Dichgans M , Fischer IR , Plesnila N , Staufenbiel M , Haass C , Snapyan M , Saghatelyan A , Tsai LH , Fischer A , Grobe K , Dimou L , Götz M. 2013 Reactive glia in the injured brain acquire stem cell properties in response to sonic hedgehog. Cell Stem Cell 12:426–439. 23561443 10.1016/j.stem.2013.01.019

[glia22850-bib-0138] Sirko S , Neitz A , Mittmann T , Horvat‐Bröcker A , von Holst A , Eysel UT , Faissner A. 2009 Focal laser‐lesions activate an endogenous population of neural stem/progenitor cells in the adult visual cortex. Brain 132:2252–2264. 19286696 10.1093/brain/awp043

[glia22850-bib-0139] Sirko S , von Holst A , Weber A , Wizenmann A , Theocharidis U , Götz M , Faissner A. 2010 Chondroitin sulfates are required for fibroblast growth factor‐2‐dependent proliferation and maintenance in neural stem cells and for epidermal growth factor‐dependent migration of their progeny. Stem Cells 28:775–787. 20087964 10.1002/stem.309

[glia22850-bib-0140] Sofroniew MV. 2014 Multiple roles for astrocytes as effectors of cytokines and inflammatory mediators. Neuroscientist 20:160–172. 24106265 10.1177/1073858413504466

[glia22850-bib-0141] Southwell DG , Nicholas CR , Basbaum AI , Stryker MP , Kriegstein AR , Rubenstein JL , Alvarez‐Buylla A. 2014 Interneurons from embryonic development to cell‐based therapy. Science 344:1240622. 24723614 10.1126/science.1240622 PMC4056344

[glia22850-bib-0142] Stahl R , Walcher T , De Juan Romero C , Pilz GA , Cappello S , Irmler M , Sanz‐Aquela JM , Beckers J , Blum R , Borrell V , Götz M. 2013 Trnp1 regulates expansion and folding of the mammalian cerebral cortex by control of radial glial fate. Cell 153:535–549. 23622239 10.1016/j.cell.2013.03.027

[glia22850-bib-0143] Suhonen JO , Peterson DA , Ray J , Gage FH. 1996 Differentiation of adult hippocampus‐derived progenitors into olfactory neurons in vivo. Nature 383:624–627. 8857538 10.1038/383624a0

[glia22850-bib-0144] Sultan KT , Brown KN , Shi SH. 2013 Production and organization of neocortical interneurons. Front Cell Neurosci 7:221. 24312011 10.3389/fncel.2013.00221 PMC3836051

[glia22850-bib-0145] Suslov ON , Kukekov VG , Ignatova TN , Steindler DA. 2002 Neural stem cell heterogeneity demonstrated by molecular phenotyping of clonal neurospheres. Proc Natl Acad Sci USA 99:14506–14511. 12381788 10.1073/pnas.212525299 PMC137913

[glia22850-bib-0146] Suzuki SO , Goldman JE. 2003 Multiple cell populations in the early postnatal subventricular zone take distinct migratory pathways: A dynamic study of glial and neuronal progenitor migration. J Neurosci 23:4240–4250. 12764112 10.1523/JNEUROSCI.23-10-04240.2003 PMC6741090

[glia22850-bib-0147] Takebayashi H , Ikenaka K. 2015. Oligodendrocyte generation during mouse development. Glia (this issue). 10.1002/glia.22863 26013243

[glia22850-bib-0148] Taverna E , Götz M , Huttner WB. 2014 The cell biology of neurogenesis: Toward an understanding of the development and evolution of the neocortex. Annu Rev Cell Dev Biol 30:465–502. 25000993 10.1146/annurev-cellbio-101011-155801

[glia22850-bib-0149] Than‐Trong E , Bally‐Cuif L. 2015. Radial glia and neural progenitors in the adult zebrafish central nervous system. Glia (this issue). 10.1002/glia.22856 25976648

[glia22850-bib-0150] Tsai HH , Li H , Fuentealba LC , Molofsky AV , Taveira‐Marques R , Zhuang H , Tenney A , Murnen AT , Fancy SP , Merkle F , Kessaris N , Alvarez‐Buylla A , Richardson WD , Rowitch DH. 2012 Regional astrocyte allocation regulates CNS synaptogenesis and repair. Science 337:358–362. 22745251 10.1126/science.1222381 PMC4059181

[glia22850-bib-0151] Vergaño‐Vera E , Díaz‐Guerra E , Rodríguez‐Traver E , Méndez‐Gómez HR , Solís O , Pignatelli J , Pickel J , Lee SH , Moratalla R , Vicario‐Abejón C. 2014 Nurr1 blocks the mitogenic effect of FGF‐2 and EGF, inducing olfactory bulb neural stem cells to adopt dopaminergic and dopaminergic‐GABAergic neuronal phenotypes. Dev Neurobiol. Doi: 10.1002/dneu.22251. 10.1002/dneu.22251 PMC6584759 25447275

[glia22850-bib-0152] Vergaño‐Vera E , Méndez‐Gómez HR , Hurtado‐Chong A , Cigudosa JC , Vicario‐Abejón C. 2009 Fibroblast growth factor‐2 increases the expression of neurogenic genes and promotes the migration and differentiation of neurons derived from transplanted neural stem/progenitor cells. Neuroscience 162:39–54. 19318120 10.1016/j.neuroscience.2009.03.033

[glia22850-bib-0153] Vicario‐Abejón C , Yusta‐Boyo MJ , Fernández‐Moreno C , de Pablo F. 2003 Locally born olfactory bulb stem cells proliferate in response to insulin‐related factors and require endogenous insulin‐like growth factor‐I for differentiation into neurons and glia. J Neurosci 23:895–906. 12574418 10.1523/JNEUROSCI.23-03-00895.2003 PMC6741904

[glia22850-bib-0154] von Holst A , Sirko S , Faissner A. 2006 The unique 473HD‐Chondroitinsulfate epitope is expressed by radial glia and involved in neural precursor cell proliferation. J Neurosci 26:4082–4094. 16611825 10.1523/JNEUROSCI.0422-06.2006 PMC6673890

[glia22850-bib-0155] Weinandy F , Ninkovic J , Götz M. 2011 Restrictions in time and space–new insights into generation of specific neuronal subtypes in the adult mammalian brain. Eur J Neurosci 33:1045–1054. 21395847 10.1111/j.1460-9568.2011.07602.x

[glia22850-bib-0156] Williams BP , Read J , Price J. 1991 The generation of neurons and oligodendrocytes from a common precursor cell. Neuron 7:685–693. 1931054 10.1016/0896-6273(91)90381-9

[glia22850-bib-0157] Wray J , Kalkan T , Smith AG. 2010 The ground state of pluripotency. Biochem Soc Trans 38:1027–1032. 20658998 10.1042/BST0381027

[glia22850-bib-0158] Wu S , Wu Y , Capecchi MR. 2006 Motoneurons and oligodendrocytes are sequentially generated from neural stem cells but do not appear to share common lineage‐restricted progenitors in vivo. Development 133:581–590. 16407399 10.1242/dev.02236

[glia22850-bib-0159] Ying QL , Wray J , Nichols J , Batlle‐Morera L , Doble B , Woodgett J , Cohen P , Smith A. 2008 The ground state of embryonic stem cell self‐renewal. Nature 453:519–523. 18497825 10.1038/nature06968 PMC5328678

[glia22850-bib-0160] Young KM , Fogarty M , Kessaris N , Richardson WD. 2007 Subventricular zone stem cells are heterogeneous with respect to their embryonic origins and neurogenic fates in the adult olfactory bulb. J Neurosci 27:8286–8296. 17670975 10.1523/JNEUROSCI.0476-07.2007 PMC6331046

[glia22850-bib-0161] Zamanian JL , Xu L , Foo LC , Nouri N , Zhou L , Giffard RG , Barres BA. 2012 Genomic analysis of reactive astrogliosis. J Neurosci 32:6391–6410. 22553043 10.1523/JNEUROSCI.6221-11.2012 PMC3480225

[glia22850-bib-0162] Zerlin M , Milosevic A , Goldman JE. 2004 Glial progenitors of the neonatal subventricular zone differentiate asynchronously, leading to spatial dispersion of glial clones and to the persistence of immature glia in the adult mammalian CNS. Dev Biol 270:200–213. 15136150 10.1016/j.ydbio.2004.02.024

[glia22850-bib-0163] Zhu X , Bergles DE , Nishiyama A. 2008 NG2 cells generate both oligodendrocytes and gray matter astrocytes. Development 135:145–157. 18045844 10.1242/dev.004895

[glia22850-bib-0164] Zhu X , Hill RA , Dietrich D , Komitova M , Suzuki R , Nishiyama A. 2011 Age‐dependent fate and lineage restriction of single NG2 cells. Development 138:745–753. 21266410 10.1242/dev.047951 PMC3026417

